# Advances of Synergistic Electrocatalysis Between Single Atoms and Nanoparticles/Clusters

**DOI:** 10.1007/s40820-024-01463-9

**Published:** 2024-07-09

**Authors:** Guanyu Luo, Min Song, Qian Zhang, Lulu An, Tao Shen, Shuang Wang, Hanyu Hu, Xiao Huang, Deli Wang

**Affiliations:** Key Laboratory of Material Chemistry for Energy Conversion and Storage (Huazhong University of Science and Technology) Ministry of Education, School of Chemistry and Chemical Engineering, Huazhong University of Science and Technology, Wuhan, 430074 People’s Republic of China

**Keywords:** Single atoms, Nanoparticles, Clusters, Synergistic composite catalysts, Synergistic effect

## Abstract

Combining single atoms with clusters or nanoparticles is an emerging tactic to design efficient electrocatalysts. Both synergy effect and high atomic utilization of active sites in the composite catalysts result in enhanced electrocatalytic performance, simultaneously provide a radical analysis of the interrelationship between structure and activity. In this review, the recent advances of single-atomic site catalysts coupled with clusters or nanoparticles are emphasized. Firstly, the synthetic strategies, characterization, dynamics and types of single atoms coupled with clusters/nanoparticles are introduced, and then the key factors controlling the structure of the composite catalysts are discussed. Next, several clean energy catalytic reactions performed over the synergistic composite catalysts are illustrated. Eventually, the encountering challenges and recommendations for the future advancement of synergistic structure in energy-transformation electrocatalysis are outlined.

## Highlights

- Fundamental principles for designing synergistic composite catalysts are reviewed.
- The synergistic effect between various active sites, promoting electrocatalytic performance in different reactions are highlighted.
- The challenges and perspectives for multiple active sites catalysts are discussed.

## Graphical abstract

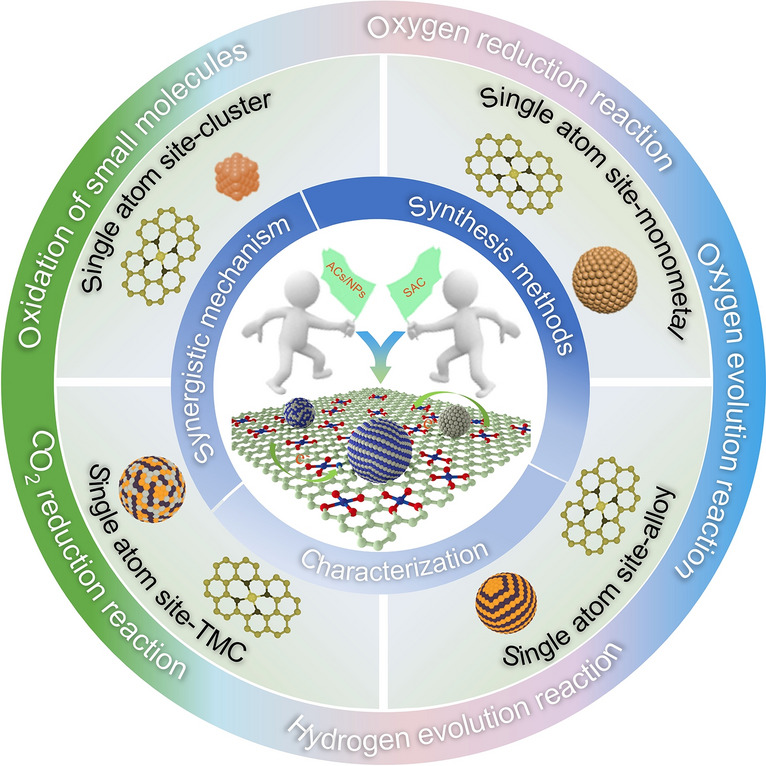

## Introduction

The development and utilization of sustainable fuels present a highly promising approach to tackle energy and environmental challenges. Electrocatalysis has gained substantial attention and research focus within the realm of energy conversion processes [1]. Over the past few decades, considerable research effort has been directed toward investigating the electrocatalytic reaction mechanism on metallic nano-catalysts, including mono-metals, alloys and transition metal compounds, etc. [2]. Compared with mono-metallic catalysts, alloy nanoparticles have exhibited significantly enhanced electrocatalytic capabilities due to the interaction of diverse elements, which optimizes electronic structures [3]. For instance, compared with pure Pt nanoparticles, PtCo nanoparticles have been observed superior electrocatalytic performance for the oxygen reduction reaction (ORR) [4, 5]. In other examples, transition metal oxide (Co_3_O_4_) also exhibits promoted oxygen evolution reaction (OER) activity [6]. Thus, nanoparticles of metal and transition metal compounds have demonstrated individual activation and optimization in electrocatalysis when they act as respective active species. Nonetheless, various configurations of atomic structure can be observed in random bimetallic alloy catalysts, posing challenges in identifying the most active coordination environment. Additionally, alloy generally consists of precious metals, resulting in high costs and restricted availability, significantly limiting their widespread applications [7]. Moreover, transition metal compounds show poor conductivity, which is detrimental to the electrochemical reaction.

Single-atom catalysts (SACs) have been investigated deeply over the past decades to upgrade the efficiency of metallic atoms usage [8–12]. Their distinct active sites, consistent activity among per catalytic center, flexible morphology, distinctive electronic structure and adjustable inherent selectivity for distinct reactions are more remarkable as compared with different sized clusters and nanoparticles [13–15]. Moreover, SACs possess potential to redetermine the correlation between catalytic performance and atomic coordination surroundings. Furthermore, the quantum size effects of them result in inconsecutive energy-level distribution and unique gaps between highest occupied molecular orbital and lowest unoccupied molecular orbital (HOMO–LUMO), offering golden chance for studying catalytic mechanism at molecular and atomic level via adjusting the coordination states [16–19]. In spite of the vast potential for applications in single-atomic catalysis, SACs encounter issues due to their straightforward structure and the absence of synergistic active sites required to exceed the inherent performance limitation in more intricate reactions. On the one hand, one type of catalytic sites possessing unique coordination structure is unfavorable for multi-elementary reaction, which is ascribed to the different adsorption energies for multiple intermediates. It may accelerate one step of a certain reaction, in spite of aiming at rate-determing step [20]. On the other hand, agglomeration usually appears as the content of metal is relatively high owing to the high surface free energy of them. Hence, the amount of metal loaded is grimly restricted in order to preserve the single atom configuration [21]. Therefore, despite the high utilization and activity per atom, the overall catalytic performance of SACs is not particularly noteworthy.

**Fig. 1:**
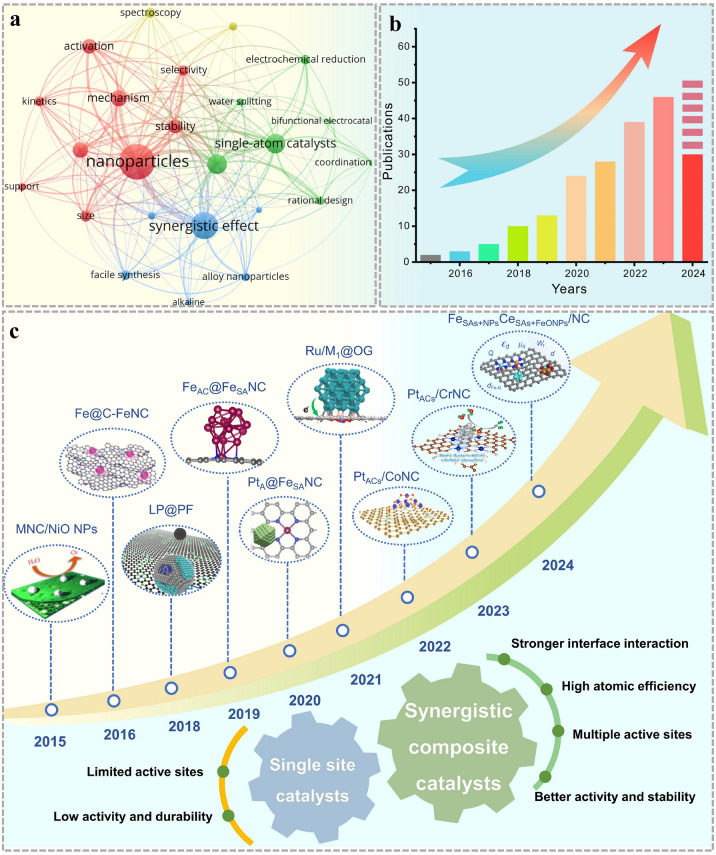
**a** The network map of keywords related to synergistic effect among nanoparticles/clusters and single atoms. **b** The number of publications based on synergistic composite catalysts. Source of the data: Web of Science; the period of publications: 2015–2024. **c** The progress of synergistic composite catalysts over the past years. MNC/NiO NPs. Reproduced with permission [22]. Copyright 2015, WILEY–VCH Verlag GmbH & Co. KGaA, Weinheim. Fe@C-FeNC. Reproduced with permission [23]. Copyright 2016, American Chemical Society. LP@PF. Reproduced with permission [25]. Copyright 2018, American Association for the Advancement of Science. Fe_AC_@Fe_SA_NC. Reproduced with permission [27]. Copyright 2019, American Chemical Society. Pt_A_@Fe_SA_NC. Reproduced with permission [28]. Copyright 2020, The Royal Society of Chemistry. Ru/M1@OG. Reproduced with permission [35]. Copyright 2021, Wiley–VCH GmbH. Pt_ACs_/CoNC. Reproduced with permission [31]. Copyright 2022, Springer Nature. Pt_ACs_/CrNC. Reproduced with permission [32]. Copyright 2023, American Chemical Society. Fe_SAs+NPs_Ce_SAs+FeO NPs_/NC. Reproduced with permission [30]. Copyright 2024, Wiley–VCH GmbH

Integrating single atoms with clusters and nanoparticles into a unified catalyst is an effective way to address these drawbacks via a synergistic catalysis. Inspired by the high activity of synergistic composite catalysts, they have been applied for various electrocatalysis. Over the past decade, the attractiveness and booming development trends about the synergistic effect among nanoparticles/clusters and single atoms has been sufficiently reflected from the dense network map of related keywords and the rapidly increasing number of publications (Fig. 1a, b). Specially, early between 2015 and 2017, Zhang and co-workers [22] took NiO as active component loaded on metal and nitrogen doped carbon sheets, to evaluate the support effect and synergistic effect of metal-nitrogen-carbon composite catalysts. Wan et al. [23] developed a catalyst, denoted as Fe@C–FeNC, consisting of Fe–N_x_ atomic center and Fe/Fe_3_C nanoparticles to examine the impact of transition metal compounds on the Fe–N_x_ coordination environment and its correlations with electrochemical performance. Subsequently mono-metal nanoparticles and clusters were introduced. In 2018, Co nanoparticles were encapsulated within nanofibers decorated with Co single atoms by Yang’s group [24]. The Co nanoparticles served as accelerator for ORR performance. Simultaneously, Chong constructed Co atoms, PtCo and Co nanoparticles into one synergistic system [25]. Lately, Zang et al. and Ao et al. set Fe nanoparticle as a regulator to modify the electronic and geometric configurations of Fe–N_4_ sites, investigating their associations with the ORR performance [26, 27]. Furthermore, the strong interaction between alloy and single atoms could assist the adsorption/desorption process to modulate various reaction performance. Liu et al. [28] confined PtFe onto Fe–N–C enhancing the ORR performance. Chong and colleagues [29] presented a novel design featuring a low-loading PtCo catalyst, employing a Pt–Co Graphene-Nitrogen nanofiber (Pt–Co–GNF) as the support structure for the PtCo core–shell nanoparticles (NPs). Moreover, the connection among nanoparticles, clusters and single-atom support was explored more deeply through DFT and machine learning [30–32]. Introducing certain metal-based nanoparticles and clusters is a viable approach to modulate the electronic structure. Meanwhile, stronger interaction can be improved through the transfer of electron between synergistic components. Establishing robust connection between single-atomic active centers and neighboring synergistic components has the potential to enhance the catalytic activity, longevity and boost reaction dynamics while upholding the advantage of exceptional atomic dispersion characteristics and stability (Fig. 1c) [26, 33–35]. However, a comprehensive review demonstrating the correlation coupled structure with activity, as well as the mechanism of synergistic effect, is still lacking.

In this review, the preparation and characterization methods of integrated system are summarized first. Combining with the result of characterization, the density functional theory (DFT) calculation is proposed to be employed to illustrate the dynamic of synergistic components, suggesting the change of electronic structure. Subsequently, the detailed classification of single atoms-nanoparticles and single atoms-clusters is provided, discussing the essential factors that govern their multiple sites structure. Furthermore, a comprehensive investigation aiming to compare, analyze and evaluate real function of these atomic-coupling configurations in energy storage and conversion applications is executed, encompassing in oxygen reduction/evolution reaction (ORR/OER), hydrogen evolution reaction (HER), carbon dioxide reduction reaction (CO_2_RR), and other reactions. Finally, the new vistas and tough challenges of this unique multiple-site synergistic effect are proposed. The objective of this review is to provide novel perspectives for readers, promoting further exploration and comprehension in the design of rational catalysts, as well as elucidating their optimal mechanisms in reactions.

## Fundamental Concepts of Single Atomic Site-Clusters/Nanoparticles Catalysts

### Synthetic Strategies

Controlled synthesis techniques, as an essential role in preparing uniformly dispersed combinations, can attain electrocatalysts with exceptional effectiveness. In the composition system involving single atoms and clusters/nanoparticles, one essential aspect of the synthesis process involves obtaining a support structure containing atomic sites, while the other involves integrating the substrate with metal clusters and nanoparticles [36, 37].

**Fig. 2:**
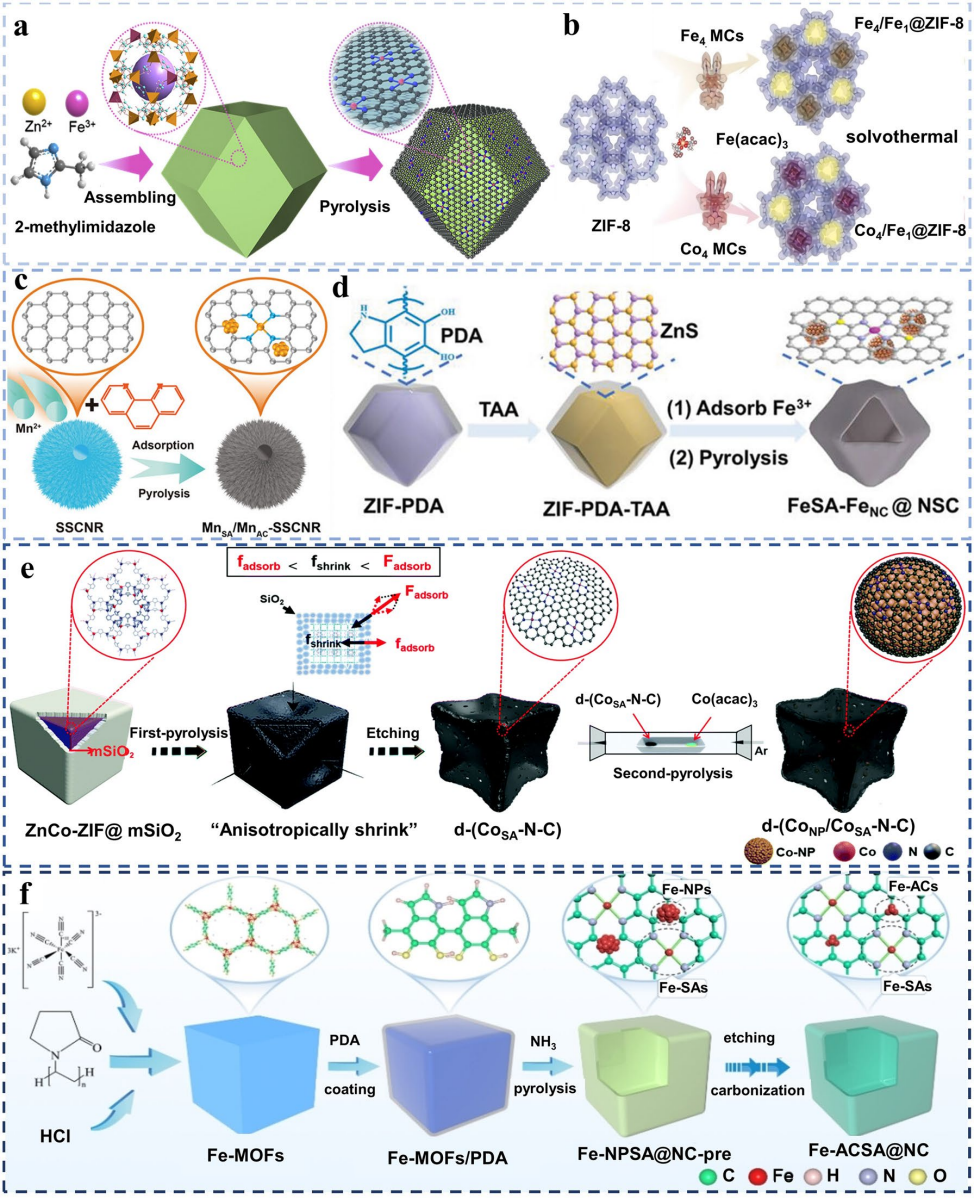
**a** Diagram of the synthetic procedures of Fe–N–C/Pd_NC_. Reproduced with permission [39]. Copyright 2022, Elsevier. **b** Scheme of synthetic process of Fe_4_/Fe_1_@ZIF-8 and Co_4_/Fe_1_@ZIF-8. Reproduced with permission [40]. Copyright 2023, Wiley–VCH GmbH. **c** Demonstration of the synthesis process of Mn_SA_/Mn_AC_-SSCNRs. Reproduced with permission [41]. Copyright 2023, Wiley–VCH GmbH. **d** Elucidation of FeSA-Fe_NC_@NSC synthetic methods. Reproduced with permission [42]. Copyright 2022, Wiley–VCH GmbH. **e** Diagram for the preparation of d-(Co_NP_/Co_SA_-N–C). Reproduced with permission [43]. Copyright 2021, The Royal Society of Chemistry. **f** Scheme of the Fe-ACSA@NC synthesis process. Reproduced with permission [44]. Copyright 2022, Wiley–VCH GmbH

For supporting substrates, wet-chemistry methods are applied widely owing to their ease of operation, affordability, and substantial potential for large-scale implementation [38]. For example, co-precipitating in mild conditions is employed in synthesizing MOFs that is one of the most commonly used precursors. Zhu et al. [39] synthesized Fe-doped ZIF-8 via a self-assembly method confining Fe in the framework (Fig. 2a). Subsequently, it could be pyrolyzed to produce Fe–N–C with rich pores serving as “cages” and high N contents. To obtain synergistic catalyst, it acted as support fixed with a secondary metal site. It indicates the promotion of ORR performance resulting from Pd_NC_-induced spin-state transition of Fe(II) from low spin to intermediate spin. Secondly, the solvothermal method is also utilized deeply. Wang and co-workers [40] performed in situ implantation of the metal precursors of tetranuclear M_4_ (M represents Co or Fe) MCs and Fe(acac)_3_ via a facile solvothermal synthesis followed by pyrolysis. The method supplied a metastable product with different size pores to confine metal crystal (Fig. 2b), limiting the size of cluster. The Fe active sites modified with M_4_ clusters exhibit excellent ORR activity, originating from the electron re-contribution behavior of Fe-N_4_ site that couples with the Co_4_ sites.

Besides, impregnation is a one-pot and straightforward technique. Coupled with this technique, high-temperature heat-treatment is indispensable. As depicted in Fig. 2c, spherical superstructure carbon nanorods (SSCNRs) derived from super structure MOF nanorods possessed a high nitrogen element content and a rich defect structure, making them suitable for loading with active sites. This characteristic allowed them to serve as an excellent platform for incorporating manganese single atoms with homogeneous atomic clusters. These Mn species were introduced via impregnation followed by a secondary high-temperature pyrolysis process. Consequently, the Mn_SA_/Mn_AC_-SSCNRs were formed and showed improved ORR activity, attributed to the synergistic interaction between Mn single atoms and Mn clusters, which optimizes and reduces energy barriers in the reaction pathway [41]. Similarly, in Fig. 2d, the vulcanization of ZIF-8 covered by polydopamine (PDA) is illustrated. Following this step, the vulcanized ZIF-8/PDA composite is employed as a substrate for Fe^3+^ adsorption through impregnation. Subsequently, this composite undergoes pyrolysis, resulting in the formation of hollow polyhedra with open nanostructures, which is beneficial to the mass transfer in reaction [42].

Furthermore, except for impregnation, atomic layer deposition (ALD) cannot be ignored. Sun and co-workers [43] synthesized d-(Co_NP_/Co_SA_–N–C) via hard template method and ALD. Initially, ZnCo–ZIF uniformly decorated with mesoporous SiO_2_ (ZnCo–ZIF@mSiO_2_) underwent preheating at 900 °C in an Ar flow (Fig. 2e). The regional stress was caused by the thermal shrinkage on the surface carbon layers, resulting in lead distortion. ALD was conducted utilizing the d-(Co_SA_–N–C) support in subsequent step. Cobalt acetylacetonate, as source of Co, was initially vaporized, then it was captured by above-mentioned support. The layer was reduced on d-(Co_SA_–N–C) carbon matrix. The concentration of CoN_4_ moiety was enhanced obviously in the carbon matrix and concurrently instituted the Co_NP_–CoN_4_ composite sites. Last but not least, “coating-pyrolysis-etching” route is widely employed for integration. Figure 2f exhibits that The Fe–MOFs were wrapped with a coating layer of polydopamine (denoted as Fe–MOFs/PDA). Subsequently, pyrolysis was carried out under NH_3_ atmosphere contributing to the enrichment of nitrogen content and get high carbonization degree. Ultimately, Fe–N–C with dispersed Fe clusters was obtained after the acid etching and second-step pyrolysis under Ar atmosphere. Acid etching dissolved nanoparticles to exposed atomic active sites, exhibiting ranking catalytic performance compared with the counterparts surrounded by nanoparticles [44].

### Characterization Methods

After integration, it is vital to determine the micro-region constructed by atoms, clusters and nanoparticles, concurrently validate the atomic-level electronic configuration, as well as coordination environment, atomic state of synergistic composite sites. Owing to the absence of crystallographic arrangement in individual metal atoms and clusters, as well as the presence of intricate interaction among different atomic-level sites, it poses great difficulties to comprehensively analyze these synergistic composite structures through the conventional characterization tools. Therefore, the techniques that are capable of characterizing interaction between them at atomic level play an indispensable role. Additionally, the coordination of various characterization techniques is required urgently, exploring the electronic structure, oxidized state, distribution at atomic-level in-depth.

**Fig. 3:**
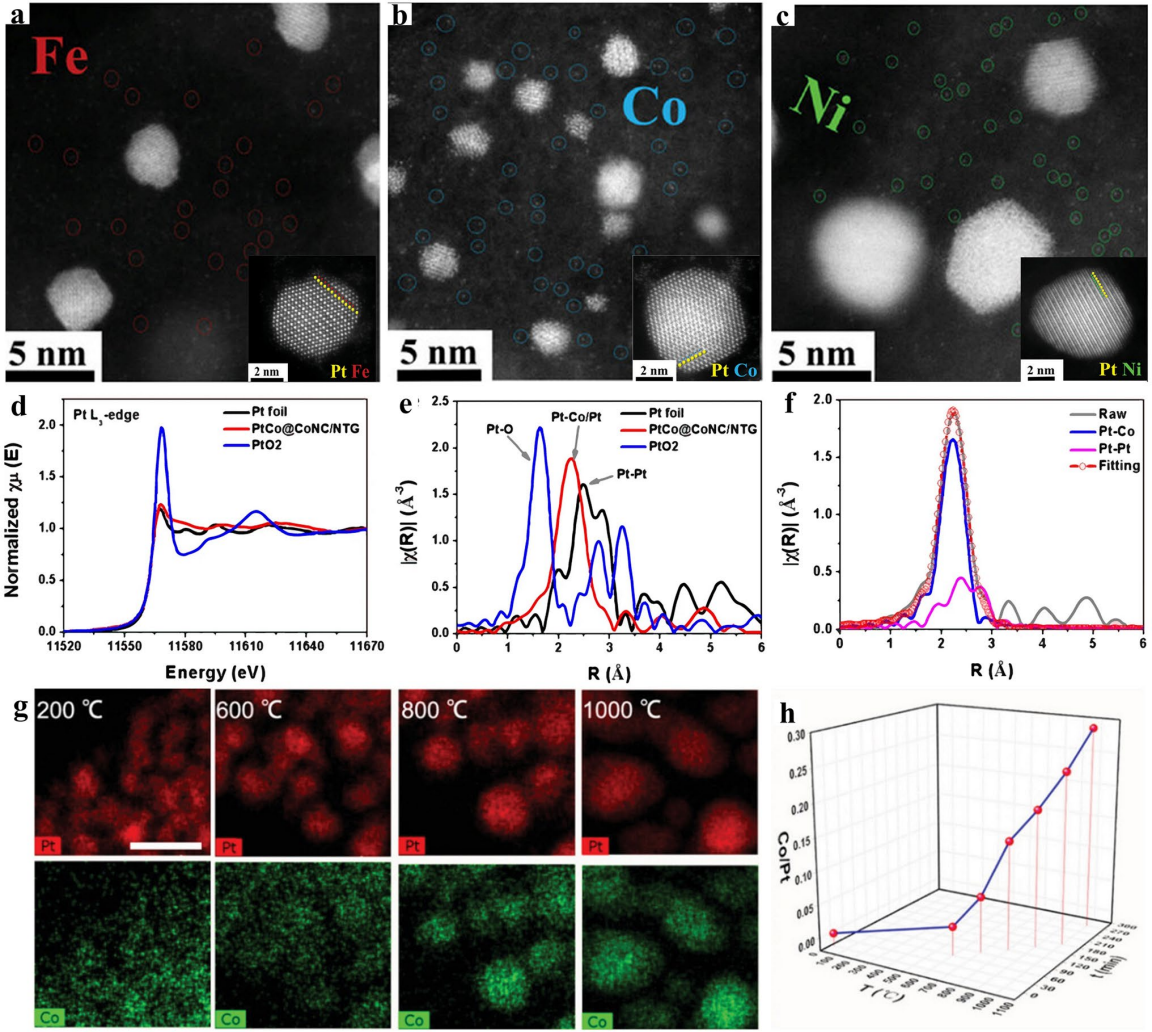
**a–c** High-resolution HAADF-STEM images for presenting metal single atoms within M–N-C aerogels and Pt-based particles locating on it, inset images presenting the ordered intermetallic structure of PtFe, PtCo, PtNi. Reproduced with permission [45]. Copyright 2023, Wiley–VCH GmbH. **d** Pt L_3_-edge XANES. **e** Fourier transforms of EXAFS spectra, and **f** the applied scattering paths of Pt L_3_-edge for PtCo@CoNC/NTG. Reproduced with permission [48]. Copyright 2022, Springer Nature. **g** EDX mapping of Pt and Co elements in 20PtCo/NC sample after calcination at increasing temperature (scale bar: 10 nm). **h** Atomic ratio of Co/Pt as functions of temperatures (T) and time. Reproduced with permission [34]. Copyright 2021, Wiley–VCH GmbH

For microscopy methods, Aberration-corrected high-angle annular dark-field scanning transmission electron microscopy (AC-HAADF-STEM) provides a direct means of observing and confirming the dispersion of single atoms and clusters/nanoparticles, making it an indispensable tool for detecting individual metal atoms and directly observing the phase of clusters/nanoparticles. Feng et al. [45] integrated metal–N–C aerogels with alloys (Fig. 3a–c). The coexistence of the alloy and single atoms could be observed. Furthermore, higher resolution had been required to prove the nanoparticles were intermetallic compounds, which definitely showed that metal atoms arranged regularly layer by layer to form super-lattice. Subsequently, the alloy process could be speculated by comparing the degree of PtFe stratification with PtCo, PtNi.

Coupled with microscopy technique, local electron energy loss spectroscopy (EELS) is indispensable to analyze existence and distribution of specific element, especially as the loading amounts of atoms is at a minute quantity. For instance, Shao et al. [46] applied it to analyze Pt–Fe–N–C, where the loading of two metallic elements was extremely low. The EELS detected strong Fe and O signals in a regular range of energy loss. As the energy loss reached higher than 2,200 eV, it revealed robust signals of platinum. This result confirmed the co-existence of Fe–N_x_ active sites, Pt–N_x_ catalytic centers, as well as PtFe nanoparticles. The above-mentioned results is a complete evidence to confirm the stable existence of various sites. Besides, Liu and co-workers [47] synthesized Pt_x_Co@SAC hybrid. The type of atomic site dispersed on support was verified, certifying that parts of platinum existed in form of single atom as analyzed by EELS.

Compared with the aforementioned methods, X-ray absorption spectroscopy provides opportunities that analyzing the coordinating numbers and environment of elements along with their chemical states. It can also give the information about electronic state of metal atoms to estimate the relationship between single atoms and cluster/nanoparticles. Through Extended X-ray absorption fine structure (EXAFS), comprehensive foresight into the interconnection between host and diverse atomic sites held on is attainable, providing an in-depth demonstration of existence form, state, and coordination environments. As depicted in Fig. 3d, zero valence state of platinum was implied by the comparable white line intensity (around 11,568 eV) was closer to Pt foil spectra, indicating that most of Pt was in the presence of metallic form. Similarly, the Fourier transformed extended X-ray absorption fine structure (FT-EXAFS) of PtCo@CoNC/NTG at Pt L_3_-edge revealed a solitary scattering peak around 2.2 Å, which was corresponding to the involvement of Pt–Pt/PtCo bonds. Notably, this peak appeared at comparatively smaller magnitude compared with Pt foil (Fig. 3e). Heteroatomic Co entering in platinum lattice changed the linear relationship of interactions. The strong interaction of Pt–Co resulted in a reduction in Pt–Pt distance compared with that of standard Pt foil, aligning well with the fitted Pt–Pt (around 2.2 Å) and Pt–Co (around 2.4 Å) signals (Fig. 3f) [48].

Besides, in situ or ex situ characterization methods are effective for understanding of material evolution process. For instance, Su and co-workers [34] investigated the Pt nanoparticles alloying process with Co on substrate by examining the distribution change in two elements at various temperatures using STEM-energy-dispersive X-ray (STEM-EDX) mappings. As portrayed by Fig. 3g, most of cobalt singe atomic site dispersed uniformly on nitrogen doped carbon substrate at 200 °C, despite a bit of them diffused into Platinum nanoparticles. However, more and more Co sites anchored on support proliferated into Pt nanoparticles to form PtCo alloy nanoparticles as elevating temperatures from 200 to 1000 °C. The size of alloy increased during the process, without the observation of Co nanoparticles. As evidenced by EDX mapping results, the measured atomic ratio of Co/Pt in PtCo (Fig. 3h) showed a gradual increase to approximately 0.3 during annealing up to 1000 °C, which was in line with the expected Pt_3_Co intermetallic stoichiometry.

Except for physical characterization, fundamental electrochemical measurement can be applied to explore the interaction between single atoms and cluster/nanoparticles. Poisoning experiments are carried out, using ethylenediaminetetraacetic acid disodium (EDTA-2Na) and potassium thiocyanide (KSCN). EDTA-2Na has been proved that it possesses selectivity in coordinating with individual metal atoms, while KSCN possesses the capability to impede the activities of both metal/alloy nanoparticles and single-atom metals [49].

Through the illustration of above samples, the significance of coordinating diverse methods has been elucidated for getting comprehensive analysis of definitive structure, as each characterization technique has its own advantages and limitations.

### Synergistic Mechanism of Single Atomic Site-Clusters/NPs

**Fig. 4:**
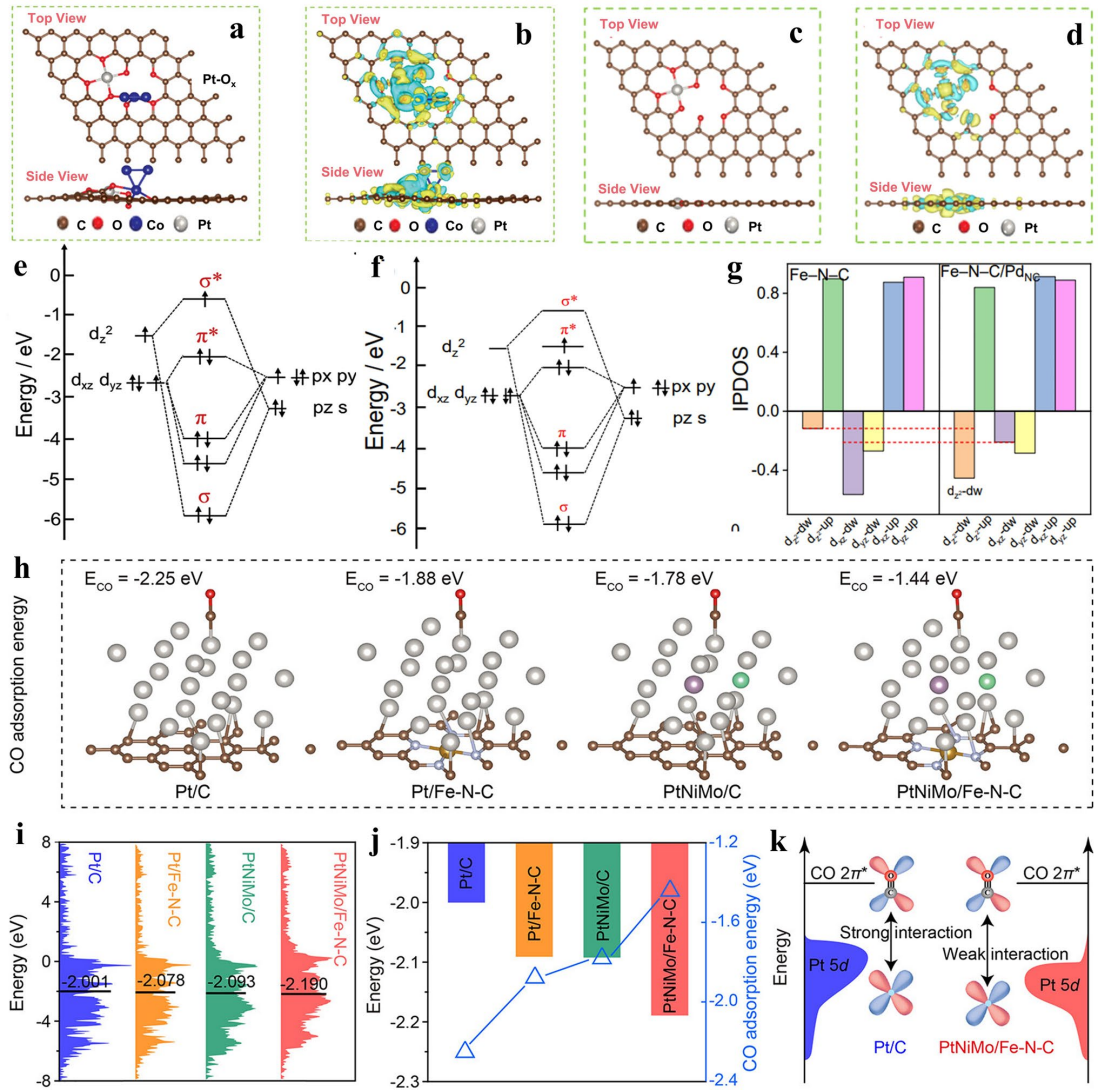
**a**–**d** Comparison analysis of atomic configuration and charge density of (Pt–O_*x*_)–(Co–O_*y*_) site and single (Pt–O_*x*_) site. (The colors yellow and cyan are used to symbolize charge accumulation and depletion, respectively, while the iso-surface's density-difference cut-off is set at a specific value: 0.035 e Å^−3^). Reproduced with permission [55]. Copyright 2022, Wiley–VCH GmbH. **e, f** Molecular orbital interactions change between OH* and Fe single atoms before and after Pd cluster loading (major connection between d_xz_, d_yz_, d_z_^2^ of Fe–N–C/Pd_NC_ and *p* orbitals in O of OH*). **g** Integrated PDOS (IPDOS) results of different orbits of Fe–N–C/Pd_NC_ and Fe–N–C. Reproduced with permission [39]. Copyright 2022, Elsevier. **h** Optimized Pt/C, Pt/Fe–N–C, PtNiMo/C, and PtNiMo/Fe–N–C models with CO adsorption energies Development of four distinct models with CO adsorption energy, showing name in diagram (brown, red, blue, gray, yellow, purple and green are on behalf of C, O, N, Pt, Fe, Mo, Ni, respectively). **i** Projected density of states exhibiting value of d-band centers (PDOS). **j** Correlation analysis of CO adsorption energy with the change in d-band centers. **k** Mechanism of interaction between CO and Pt-based active sites. Reproduced with permission [56]. Copyright 2023, American Chemical Society

The essence of investigating synergistic mechanism is to clarify the interaction between single atoms and cluster/nanoparticles. In addition to characterization, density functional theory calculation (DFT) is significant to develop in parallel with experiments [50–54]. It is capable of acquiring the charge distribution, adsorption energies, and elucidating reaction mechanism. In particular, for the synergistic composite catalysts constructing single atom sites with clusters and nanoparticles, DFT can be applied to investigate the synergistic effect. Specially, Jiao et al. [55] utilized DFT to comprehend the synergistic mechanism between Pt–O_x_ and Co–O_y_. Based on the results of characterization, (Pt–O_x_)-(Co–O_y_) structure was built, which was perfectly nonbonding at atomic scale (Fig. 4a, b). As a comparative model, the defective carbon substrates were employed to simulate a distinct Pt–O_x_ structure, exclusively loaded with it (Fig. 4c, d). A noticeable shift in charge density revealed a decrease in Pt charge within the Pt–O_x_ site, while the presence of numerous non-localized charges was observed in close proximity to oxygen atoms. Therefore, the variation of charge density is the primary and fundamental manifestation after constructing them in a unified system.

Secondly, the spin-state transition of atoms can be altered via an integration strategy. Zhu et al. [39] achieved that Pd clusters was dispersed well on Fe–N–C. The spin-state transition of Fe–N_4_ sites was change from low-spin (LS) to intermediate-spin (MS) through the introduction of Pd_NC_. In comparison to Fe–N–C, the presence of a higher spin state at the Fe site in Fe–N–C/Pd_NC_ resulted in an expanded spin-related pathway, thereby facilitating charge transport. Considering the perpendicular orientation of *d*_z_^2^ in relation to the Fe–N–C plane, the transition from an unoccupied *d*_z_^2^ orbital in low-spin Fe(II) configuration to a partially occupied one in intermediate-spin configuration, effectively modulated the extent of orbital overlap with oxygen intermediates (Fig. 4e, f). Moreover, the incorporation with Pd_NC_ facilitated the transfer of electrons from Fe single atoms to Pd_NC_, thereby inducing significant reorganization of electron distribution in Fe 3*d*-orbital. This phenomenon could be primarily attributed to the pronounced enhancement of Fe *d*_z_^2^ orbital spin state, as evidenced by meticulous orbital analysis (Fig. 4g).

Besides, Hu et al. [56] conducted DFT calculations to investigate the influence of CO adsorption on the structure constructed according to different effect, as it played a crucial role in determining the electrocatalysts’ ability to withstand CO-tolerance. The study involved the construction of four distinct models, including Pt/C, Pt/Fe–N–C, PtNiMo/C and PtNiMo/Fe–N–C. The second sample was built following strong metal-support interaction (SMSI) effect. Third one was constructed corresponding to alloying effect. The sample containing Fe single atom and alloy combined alloying with SMSI effect (Fig. 4h). By effectively harnessing the synergistic advantages of alloying and SMSI effect, PtNiMo/Fe–N–C demonstrated a remarkably diminished CO adsorption energy of − 1.44 eV, indicating a synergistic attenuation of CO adsorption and assisting CO poisoning in mitigating on Pt sites during hydrogen oxidation reaction (HOR). Subsequently, the Pt d-band centers were calculated. Through comparison, it was observed that the integration of PtNiMo alloy with substrate full of Fe single atoms yielded a significantly reduced value of the d-band center (− 2.190 eV), emphasizing the potential utilization of synergistic function caused by both alloying and SMSI effect to modulate Pt d-band centers effectively (Fig. 4i). As exemplified by Fig. 4j, the location of the d-band center exhibited a significant correlation with the CO adsorption energies. Moreover, from the perspective of mechanism, the PtNiMo/Fe–N–C catalyst, with its d-band center positioned at the lowest level, effectively mitigated electron back donation from Pt 5*d* to CO 2π* (Fig. 4k). This theoretical finding suggests that it demonstrates superior resistance against CO in HOR.

Although the impact of the interaction between synergistic sites on their own is manifested through alterations in electronic structure, there are different functions when synergistic sites work for various reaction. It can be classified into four types. The first type is that the main catalytic reaction is promoted on one active species by nearby shielding, which suppresses a competitive reaction on another active species. This kind of catalysts are usually employed in oxidation of micro-molecule. The second type involves only one active species serving as the reaction site, while the adjacent modifier regulates its electronic structure. The third type involves that the active species independently catalyze one or multiple electron-transferring steps in catalytic reactions involving multiple electrons, synergizing the overall catalytic process within a specific reaction route, typically in CO_2_RR. The catalysts of the fourth type can be referred to as bifunctional catalysts, ascribed that two active species catalyze two contrast catalytic reactions at anode and cathode in devices, respectively, synergizing in the overall catalytic process. The enhancement of this type is often observed at the device level, such as proton exchange membrane water electrolysis.

Ultimately, optimizing integration introduces new active sites and leads to the modulation of electronic structure, which can promote the reaction kinetics in various way. DFT calculation is an effective method to demonstrate electronic configuration definitely and the dynamics of synergy through the simulation in reaction processes.

## Synergistic Components of Single Atomic Site Catalysts

### Single Atomic Site-Mono-Metal Nanoparticle

**Fig. 5:**
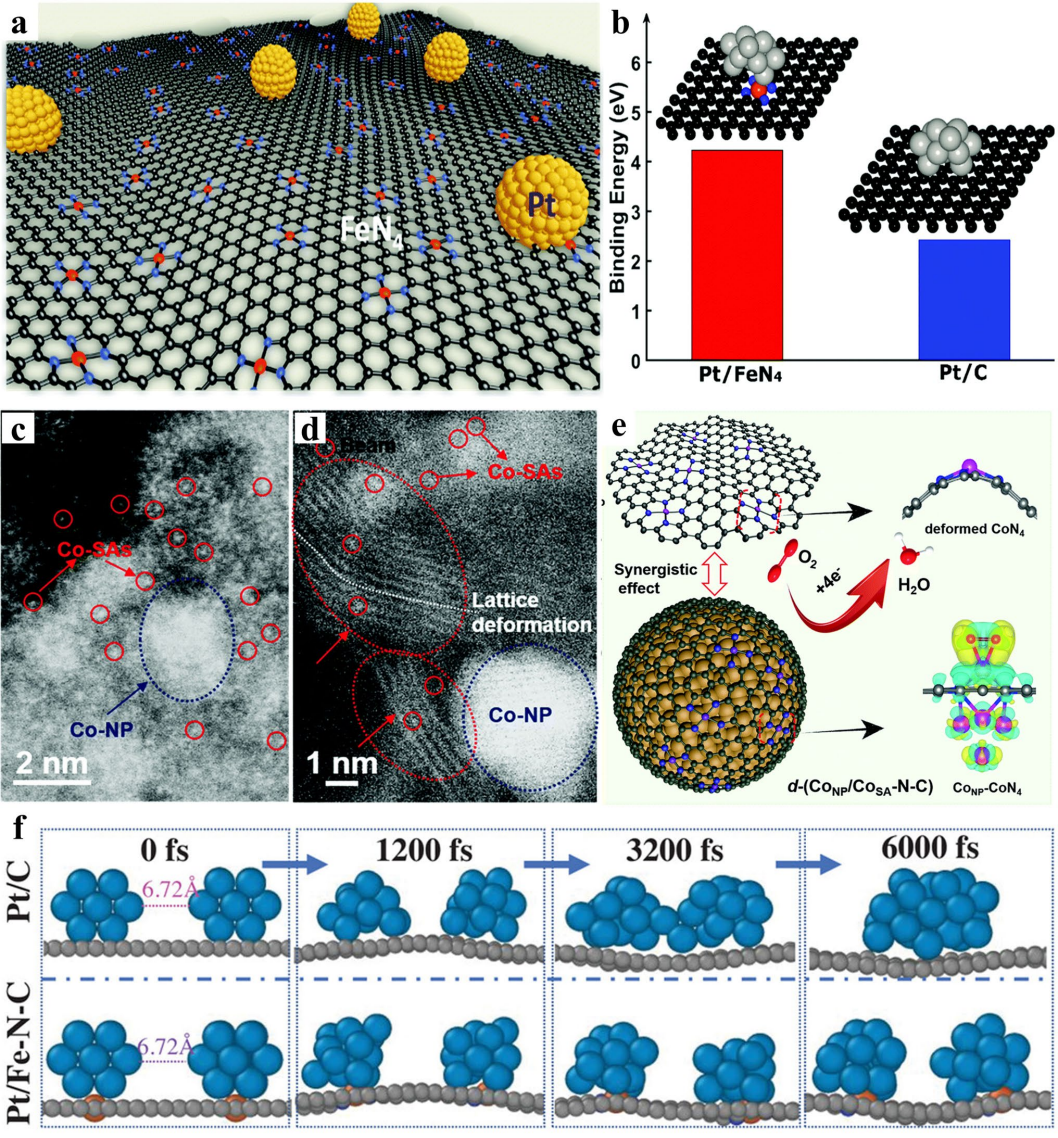
**a** Abstract blueprint of Pt/FeN_4_–C catalysts, revealing the simultaneous presence of Pt-based nanoparticles and Fe–N_4_ single atomic sites. **b** Model of two samples, and Comparison of the calculated binding energies of Pt/FeN_4_ and FeN_4_, illustrating the contribution of PGM-free single site to the stronger interaction. Reproduced with permission [68]. Copyright 2021, The Royal Society of Chemistry. **c, d** HAADF-STEM images of Co_NP_/Co_SA_-N–C showing coexistence of different size active sites. **e** Manifesting about ORR mechanism at Co_NP_/Co_SA_–N–C. Reproduced with permission [43]. Copyright 2021, The Royal Society of Chemistry. **f** Comparison of molecular dynamics results showing the agglomeration tendency of two Pt_13_ particles loaded on the carrier at room temperature, establishing the vital function of single atom in SMSI effect. Reproduced with permission [45]. Copyright 2023, Wiley–VCH GmbH

SACs often exhibit inadequate reaction activity. Thus, integrating other metal nanoparticles with SACs is regarded as a novel strategy to enhance the reaction performance through modulating the electronic structure [57–62]. It can be categorized into two types. One type lies in the fact that single atoms are integrated with precious metal nanoparticles, the other is combining single atoms with transition metal nanoparticles [63–67]. For the former type, Wu et al. [68] integrated Pt with Fe–N_4_ single metal active site (Fig. 5a). The interplay between decorated support and Pt enhanced the value of binding energy, from 2.43 eV of Pt/C to 4.23 eV of Pt/FeN_4_–C, which indicated the effective function of decoration for stronger interaction (Fig. 5b). For other precious metal, Lin and co-workers [69] reported a pyrolysis strategy driven by 1-naphthylamine to conform Fe–N_3_ SAs into Pd nanocrystals with sizes below 5 nm, which were incorporated in N-doped porous carbon nanobelts. The computation model involving FeN_3_C–Pd(111) revealed that the carbon carrier loaded FeN_3_ SAs possess higher affinity to Pd(111), which resulted in localized deformation of the surface Pd layer. According to the DFT, It was evident that FeN_3_C SAs shifted the d-band center of Pd near the Fermi level, resulting in an increased DOS of FeN_3_C–Pd(111) near the Fermi level. The enhanced interaction between FeN_3_C and Pd was beneficial to the reduction in rate-determining step free energy.

Sun et al. [43] constructed a composite site which contained Co nanoparticles and Co–N_4_ (Fig. 5c). The catalyst surface underwent high-resolution imaging, which yielded further insights into the degree of curvature and distortion observed in the outer carbon layers. Meanwhile, Numerous Co single atoms were extensively scattered across the external layers, leading to distorted configurations of the CoN_4_ structure within these outer layers (Fig. 5d). Subsequently, the DFT results presented that the integration of deformed CoN_4_ and Co nanoparticles proved to be efficacious in achieving a complete density restoration (Fig. 5e). Additionally, Zhou et al. [70] combined highly dispersed Fe atoms with Co nanoparticles on N-doped porous carbon supports. The interaction of Co nanoparticles and Fe single atoms promoted the electron redistribution effectively, modulating the adsorption of oxygen intermediates. The change in electron structure contributes to the superior oxygen electrocatalysis performance. Thus, tailoring the electron distribution by constructing nanoparticles and single atom into one system is useful.

Moreover, strong metal-support interactions between single atom loaded on substrate and nanoparticles can prevent the aggregation while pyrolyzing. Concurrently, it is plausible that the exceptional durability and effectiveness of Pt metal/M–N–C catalysts could be attributed to SMSI effect. Feng et al. [45] utilized molecular theoretical simulation and calculation to verify the effect. As represented in Fig. 5f, the two Pt_13_ particles were at a distance of 6.72 Å initially. With time going, the two particles on the carbon substrate without any other preprocessing, gradually became convergent. Owing to the absence of limiting effect, complete aggregation into a single larger particle was exhibited at 6000 fs. In contrast, obvious tendency for agglomeration was scarcely visualized in the particles adsorpted by the Fe sites in Fe–N–C. The results suggested that the strong attraction existed between Fe sites and Pt particles, effectively preventing their aggregation, maintaining them at a controlled size (approximately 3 nm consistent with experiments) and maximizing atom utilization, for the sake of obtain higher ORR performance.

To improve the stability of catalysts in reaction, Shao and co-workers [59] applied Fe and N co-doped carbon (Fe–N–C) as support for Pt nanoparticles. It demonstrated excellent durability, with 99% and 71% ECSA retention after 10,000 cycles under 0.6–1.0 V in acidic and alkaline electrolytes, respectively. This result is much better than commercial Pt/C and Pt/N–C with 78% and 53% preservation, respectively, under the same conditions. Meanwhile, PDOS of Pt in Pt/Fe–N–C and other two samples to analyze was compared to elucidate the influence of support decorated with Fe–N_4_ sites, the d-band center of Pt exhibited a downward shift about 0.12 eV. The phenomenon of negative shift was effective to weaken the adsorption strength of Pt/Fe–N–C with the oxygen intermediates (O*), promoting the catalytic processes.

From the analysis, it is obvious that the single atom sites on the substrate have a key influence in strengthening metal-support interactions, which is significant for the stability of catalysts, as well as electronic structure can be modulated effectively, thereby regulating the adsorption strength of intermediates and promoting the catalytic performance.

### Single Atomic Site-Alloy Nanoparticle

Compared with mono-metal nanoparticles, alloying with additional metal induces compressive strain and increases electron density. The strong electronic interaction between another metal component and Pt plays a significant role in optimization of electron structure, atom ensemble configurations and promotion of electrochemical performance. Besides, as alloy transformed into an intermetallic phase, intermetallics acquire enhanced stability, ascribed to strong Pt(5d)–M(3d) coupling effect [71]. Thus, integrating alloy with single atoms will be an effective way to obtain more efficient electrocatalysts [72–77]. As pioneers, Liu et al. [28] reported a synergistic composite catalyst combining nitrogen doped carbon derived from Zn-ZIF with Fe atoms and in situ generated core–shell PtFe nanoparticles containing ordered structure (named as Pt_A_@Fe_SA_–N–C). According to the AC–TEM, the PtFe intermetallic was encompassed by heavily distributed Fe and residual Zn after vaporization anchored on carrier. Theoretical calculations indicated that the rate-determining step for single atom sites and synergistic sites involved the detachment of the OH* intermediate. The onset potential of synergistic model was highest (1.01 V) either, indicating improved catalytic activity due to the synergistic function of Pt and Fe–N_4_. This phenomenon was attributed to the higher electro-negativity of the Pt atom compared with the Fe atom, causing an electron transfer.

**Fig. 6:**
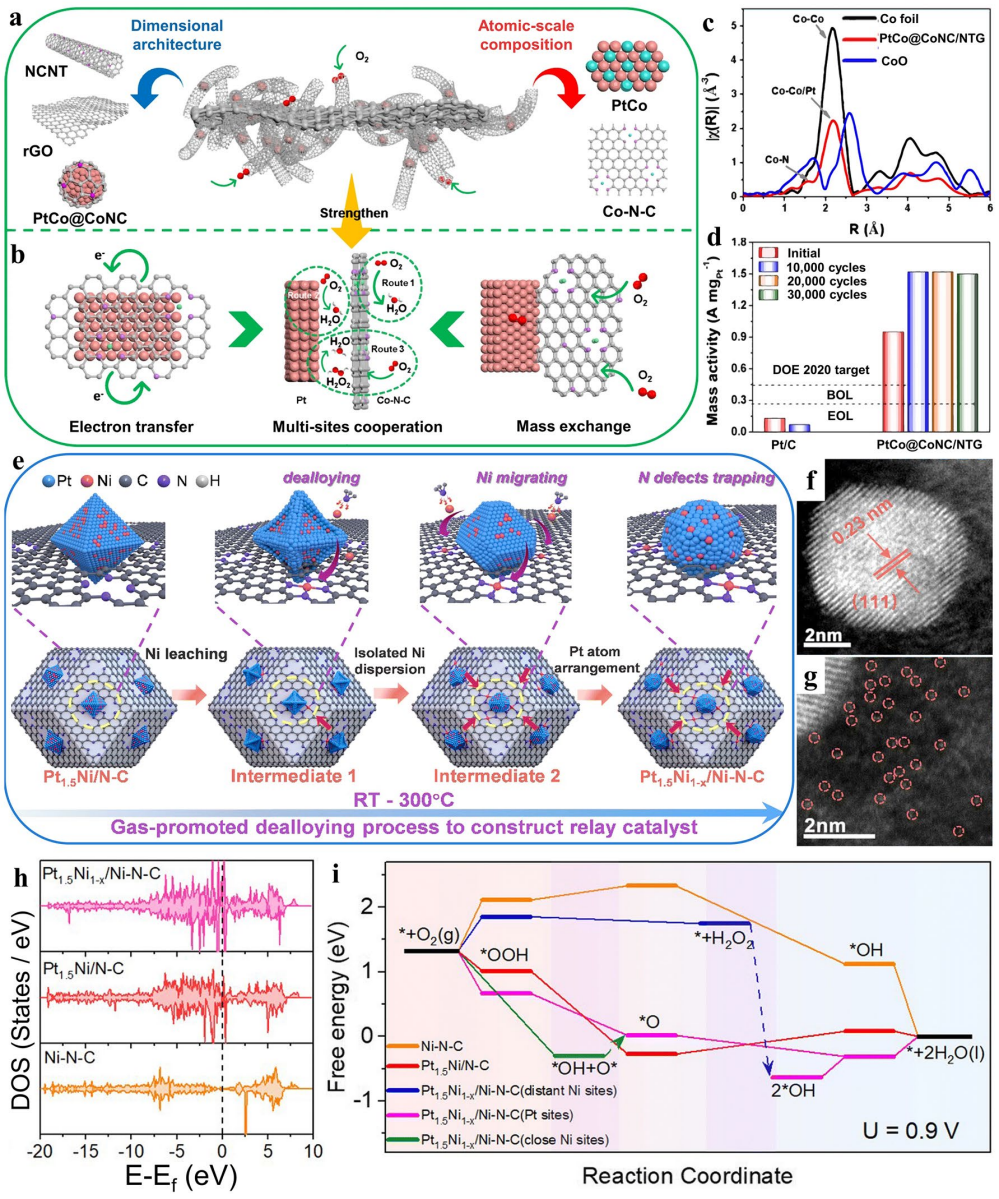
**a** Abstract blueprint of construction for PtCo@CoNC/NTG. **b** Synergy of various components for promoting the ORR process. **c** Fourier transforms of EXAFS spectra of PtCo@CoNC/NTG. **d** ORR performance comparison of Pt/C and PtCo@CoNC/NTG. Reproduced with permission [48]. Copyright 2022, Springer Nature. **e** Conceptual graphic for the preparation of relay catalyst Pt_1.5_Ni_1-x_/Ni–N–C. **f, g** AC-HAADF-STEM image showing the coexistence of two sites. **h** DOS and the corresponding d band centers of Pt_1.5_Ni_1-x_/Ni–N–C, Pt_1.5_Ni/N–C and Ni–N–C. Black dashed line: Fermi-level. Purple line: d band center. **i** ORR free energy scheme at 0.9 V of Ni–N–C, Pt_1.5_Ni/ N–C, Pt_1.5_Ni_1-x_/Ni–N–C with distinct distance to Ni sites. Reproduced with permission [78]. Copyright 2023, The Royal Society of Chemistry

In another example, Xia and co-workers [48] presented a highly effective integrated electrocatalyst comprised of platinum and nanocarbon. The design concept regarding the various influence and function of different sites was demonstrated in Fig. 6a. It was accomplished through a multiscale approach, fabricating nanocarbon supports at the architectural level with PtCo and Co atomic components at the atomic level. Figure 6c exhibits that PtCo@CoNC/NTG possessed only one scattering peak at approximately ~ 2.2 Å. This peak was ascribed to involvement of Pt–Co, with a lower magnitude in comparison to Pt foil. These results indicated the co-existence of multi-active sites. Meanwhile the collaboration among multi-sites optimized the reaction pathways, accelerated electron transfer, as well as mass exchange (Fig. 6b). Consequently, the synergistic composite catalyst exhibited remarkable ORR activity. The initial activity reached 1.52A mg_Pt_^−1^ at the 10,000th cycle, while experiencing only a 1.3% attenuation at the end-of-life (EOL). Over the subsequent cycles (Fig. 6d).

Moreover, Wu et al. [78] proposed a dealloying process making use of single atom on carrier to create composite electrocatalysts comprising PtNi nanocrystals and densely isolated Ni sites (Fig. 6e). Following structural optimization, a greater variety of active sites emerged. The alloy nanoparticles and single atoms could be observed obviously (Fig. 6f, g). The density of states (DOS) illustrated in Fig. 6h showed that Ni–N–C represented extreme absence of electronic states. On the contrary, Pt_1.5_Ni_1-x_/Ni–N–C and Pt_1.5_Ni/N–C catalysts had exhibited an increased DOS toward the Fermi-level, which implied a greater charge transfer ability with the introduction of Pt. Furthermore, the highest abundance of electronic density of Pt_1.5_Ni_1-x_/Ni–N–C intimated potential synergy between PtNi and Ni single atom sites, contributing to improved electrocatalytic performance. Correspondingly, the PtNi displayed significantly lower free energy change than Ni–N–C sites (Fig. 6i), indicating a stronger inclination toward the four-electron ORR on this kind synergistic composite catalyst. In this way, integrating alloy and single-atom sites into one system is an effective method to modulate electronic structure and diminish the usage of precious metal [29, 79].

In additional to these Pt-based alloy, transition-metal based-alloy is a synergistic component of single atoms. Zhang et al. [80] developed N-doped porous carbon janus-like frameworks as support for atomic Co/Ni atomic sites and Co–Ni alloy. It exhibits a low overvoltage between ORR and OER, with 0.78 V. The superior performance is originated from topological structure and synergistic effect of this composite synergistic catalysts.

For this special synergistic catalysts, the electronic structure is modulated by alloying, and then synergistic effect between alloy and atomic sites on support constructed electronic interaction more deeply. It is essential for regulating intermediate adsorption strength to get enhanced catalytic performance.

### Single Atomic Site-Transition Metal Compounds (TMCs) Nanoparticles

Transition metal compounds, which exhibit limitations in terms of the presence of catalytically active sites at low concentrations, tend to undergo aggregation and do not confer a competitive advantage for electrocatalytic applications. After integrating with single atoms, this synergistic catalyst was constructed without precious metal, which was beneficial for application in various fields [81–86].

**Fig. 7:**
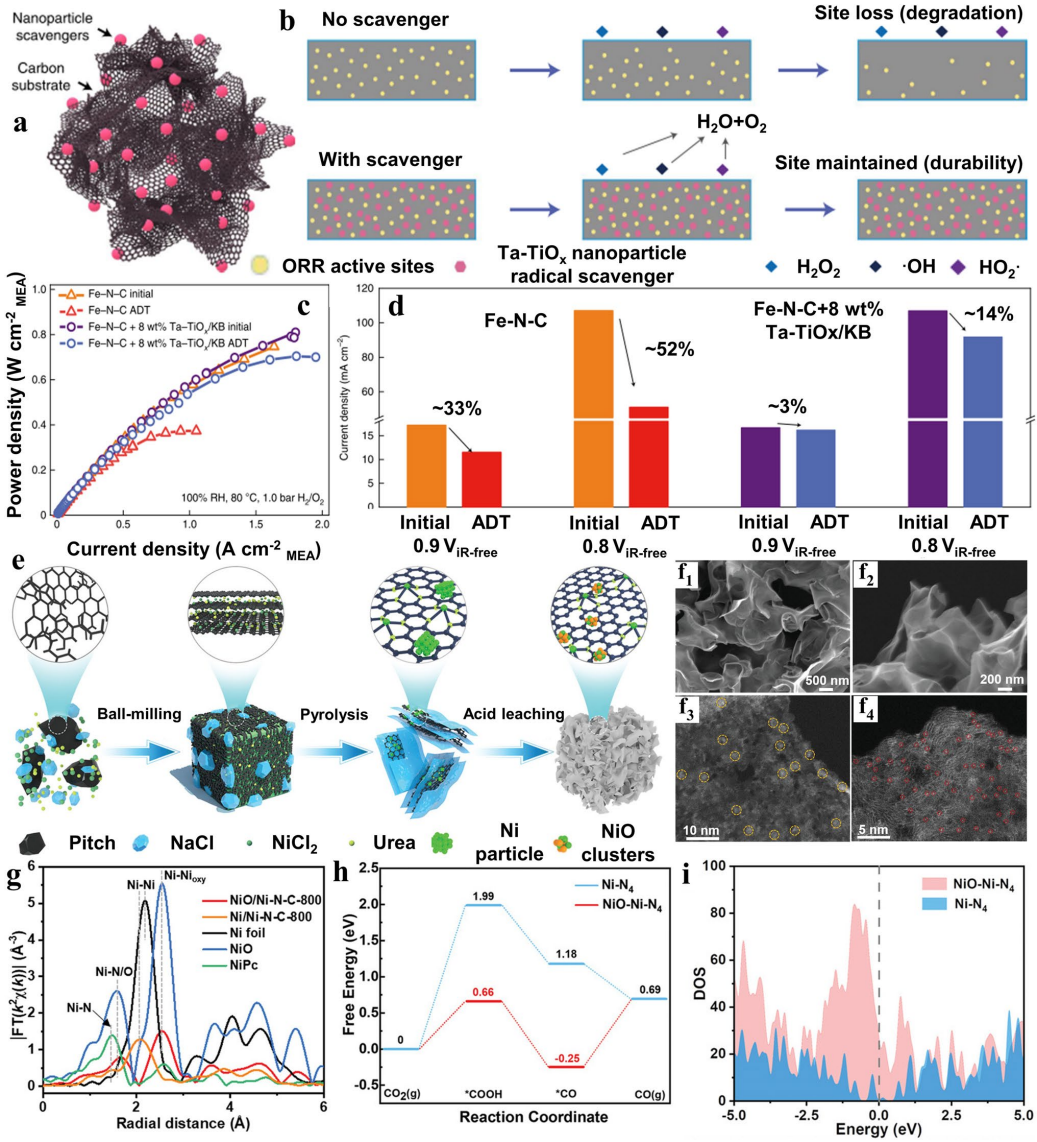
**a** Schematic depiction of Ta–TiO_x_ nanoparticles loaded on graphene as catalytic scavengers. **b** Comparison diagram of Fe–N–C durability in the ORR process with scavengers showing protection for active sties and without scavengers. **c** Comparison of fuel cell performance before and after the ADT. **d** Durability at different voltage comparison for cells with and without Ta–TiO_x_/KB after the ADT, illustrating the protective effect of Ta–TiO_x_ scavengers. Reproduced with permission [87]. Copyright 2022, Springer Nature. **e** Synthesis processes of NiO/Ni–N–C-800 catalysts. **f**_**1**_**, f**_**2**_ SEM images of stripped pitch loading NiO and Ni single active sites. **f**_**3**_**, f**_**4**_ HAADF-STEM images of NiO/Ni–N–C-800, exhibiting NiO cluster and Ni single atoms marked with two colors. **g** Fourier transform of the EXAFS spectra of NiO/Ni–N–C-800, as well as other samples. **h** Calculated free-energy diagrams for transformation of CO_2_ to CO over single sites and synergistic catalytic sites. **i** Calculated DOS for Ni–N_4_ and NiO–Ni–N_4_ catalytic sites. Reproduced with permission [89]. Copyright 2022, Wiley–VCH GmbH

Subsequently, Hu and co-workers [87] incorporated Ta–TiO_x_ nanoparticles with Fe–N–C to address degradation issue (Fig. 7a). A comparison of Fe–N–C and synergistic composite catalysts behavior concerning OH*, HO_2_*, as well as H_2_O_2_ is depicted in Fig. 7b. The durability enhancement of Fe single sites was proved via comparing in the presence and absence of Ta–TiO_x_/KB scavengers. In the absence of scavengers, the sharp decrease in ORR efficiency can be attributed to the exhaustion of catalytic sites. By introducing Ta–TiO_x_ scavengers, the HO*, as well as H_2_O_2_ generated during the 2e^−^ oxygen reduction pathway were actively decomposed. Corresponding to the fuel cell performance, the electrode, employing the Ta–TiO_x_/KB scavengers, established optimal performance with a current density of 0.63 A cm^−2^ at 0.6 V and a maximum power of 700 mW cm^−2^ (Fig. 7c). According to the comparison in Fig. 7d, the cell lacking Ta–TiO_x_ featured a considerable decline in current density following the durability test, whereas the Ta–TiO_x_-equipped cell displayed only a negligible decay. These results indicated that the enhancement of durability could be attributed to the vital contribution of Ta–TiO_x_ scavengers reasonably. Additionally, Chen et al. [88] described an approach for synthesizing N, P co-doped carbon frameworks (NPCFs) incorporating loaded Fe single atoms and Fe_2_P nanoparticles (named as Fe SAs–Fe_2_P NPs/NPCFs). The highlight of this technique was in situ doping-adsorption phosphatization. The typical sample showed that many nanoparticles (Fe_2_P NPs) were surrounded by highly dispersed single atoms, with the optimal catalytic performance. This finding elucidated that introduction of phosphide was functional to improve single atomic catalysis either. The synergistic effect between them was able to weaken the adsorption of oxygen containing intermediates and supply new reaction pathway.

Moreover, He and colleague [89] constructed NiO clusters with Ni–N–C SACs (Fig. 7e). As depicted in Fig. 7f_1_ and f_2_ the substrate exhibited a well-defined three-dimensional (3D) framework structure featuring highly porous interconnected thin sheets. Simultaneously, the Ni SA and NiO clusters dispersed uniformly on it shown in Fig. 7f_3_ and f_4_. Figure 7g depicted the primary peak of NiO/Ni–N–C-800 at around 1.66 Å, which could be ascribed to Ni–N/O. It was worth noting that the absence of Ni–Ni, corresponding to nickel metal foil. Additionally, from the other main peak at 2.6 Å, the major component of Ni existing in NiO/Ni–N–C-800 was NiO clusters. The remaining Ni was presented as single active sites coordinated with nitrogen and oxygen atoms. With the introduction of NiO, it was easier to format COOH* and CO* with lower energy on synergistic composite sites (Fig. 7h). Figure 7i illustrated that the Ni 3*d* orbitals of NiO–Ni–N_4_ demonstrated significantly greater density of states than that of Ni–N_4_ in close proximity to Fermi-level.

Consequently, constructing TMCs and single atom into a single system is functional. It can be divided into two types. One of them acts as protective agent for active sites, the other one acts as another active sites. Otherwise, it is an effective way to reduce catalyst cost.

### Single Atomic Site-Clusters

Compared with nanoparticles, smaller clusters possess diverse coordination structures and short interacting distance, which can make electronic interaction stronger and promote the electronic structure modulation [90–96]. As an example, Xu et al. [97] developed a carbon-supported atomically dispersed Pt catalyst containing both individual Pt atoms and clusters. It presented high activity (1148 mW cm^−2^) and durability for HOR. It could be attributed to the mechanism of a synergetic effect between Pt single atoms and the neighboring clusters. Meanwhile, some scientists set nanoclusters as modifier. Zhang et al. [98] engineered Fe–N_4_ electronic structure with adjacent Co–N_2_C_2_ and Co sub-nanoclusters to optimize the activity of Fe–N_4_ active sites.

**Fig. 8:**
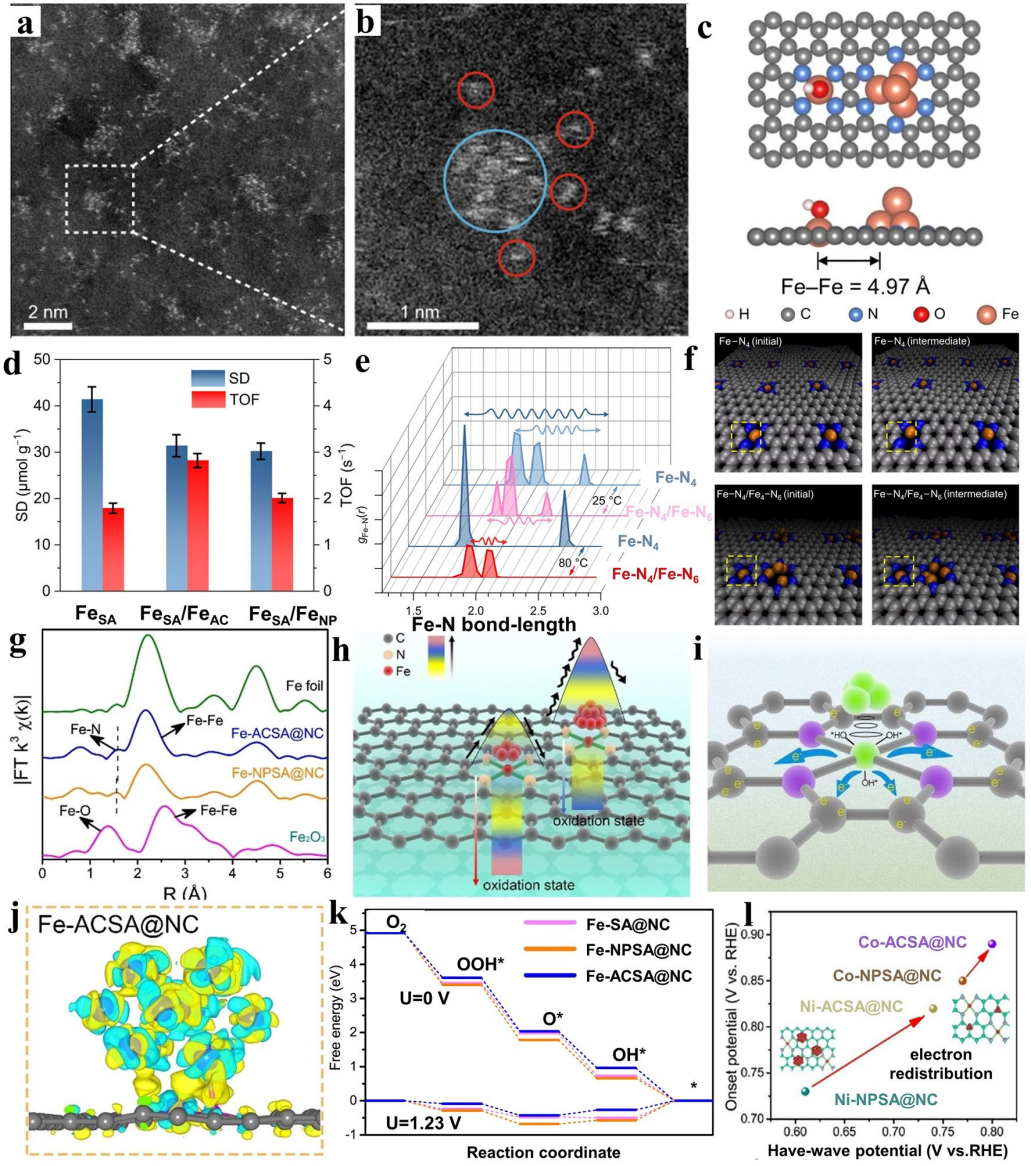
**a, b** HAADF-STEM image showing overall distribution, coupled with area enlargement in image showing an micro-synergistic region comprising of cluster and its satellite single atoms. **c** Model structure of Fe–N_4_/Fe_4_–N_6_ showing distance between two sites with a naturally formed OH ligand. **d** SD and TOF conducting on Fe-N_4_ sites of the specified Fe–N–C. The error bars depict the standard deviation obtained from three distinct measurements conducted. **e** Fe–N radical distribution function profiles of the Fe–N_4_ moiety in the models of bare Fe–N_4_ and Fe–N_4_/Fe_4_–N_6_ at 25 and 80 °C. Wavy arrows are employed to denote the extent of fluctuation in the bond length between Fe and N. **f** Images demonstrating the result of molecule dynamic simulations of Fe–N_4_ and Fe–N_4_/Fe_4_–N_6_ at 80 °C. Reproduced with permission [99]. Copyright 2022, Springer Nature. **g** Fourier transform k^3^ EXAFS spectra. **h** Diagram depiction of the relationship between ORR efficiency and change of oxidation state in Fe-ACSA@NC and its counterparts. **i** Elucidation of the electron redistribution with the introduction of metal clusters. **j** Differential charge density of Fe-ACSA@NC. **k** Diagram illustrating the energy landscape of the oxygen reduction reaction for all samples at 1.23 and 0 V. RHE. **l** Comparison of ORR indexes among other two kind of synergistic composite catalysts containing different transition metal elements. Reproduced with permission [44]. Copyright 2022, Wiley–VCH GmbH

Besides, Shui and co-workers [99] fabricated Fe clusters accompanied by satellite Fe–N_4_ sites on porous carbon matrix. The simultaneous presence of Fe single atoms and few-atomic clusters was evident on carrier, as illustrated in Fig. 8a. The enlarged depiction in Fig. 8b offered a clearer view, illustrating several iron atoms closely surrounding a cluster at a distance of < 0.5 nm. The close proximity between sites enabled the swift transfer of electrons among distinct sized active sites. Subsequently, DFT calculations were conducted referring to the results of characterization, highlighting the distance of 4.97 Å between Fe clusters and their satellite single atoms (Fig. 8c). The presence of OH ligand significantly enhanced the binding affinity of the Fe–N_4_ site toward the oxygen intermediates involved in ORR, thereby considerably diminishing the energy barrier. Thus, it indicated that the clusters mainly acted as an activity booster. The results were in line with experiments that is Fe_SA_/Fe_AC_-2DNPC exhibited a TOF enhancement reaching approximately 60% at Fe–N_4_ site compared with Fe_SA_–2DNPC (Fig. 8d). Additionally, the durability performance of active sites was explored through molecular dynamics (MD) simulations, focusing on the fluctuation of bond lengths. As depicted in Fig. 8e, the stability of Fe–N_4_ site in Fe–N_4_/Fe_4_–N_6_ was found to be higher than that of an isolated Fe–N_4_ site at 25 °C. While elevating to 80 °C, the Fe–N bond-length distribution increased in Fe‐N_4_ but remained narrow in Fe–N_4_/Fe_4_–N_6_. Besides, the iron clusters additionally induced a pinning effect, effectively suppressing the thermal oscillations of the satellite Fe–N_4_ sites at 80 °C (Fig. 8f), thereby reducing their susceptibility to demetalation.

In another example, Peng et al. [44] applied Fe metal atomic clusters (AC) to functionalize Fe single-atoms (SA) and discussed the generality for other metal. The coordination structure data could be supplied by Fourier transform k^3^ EXAFS Spectra and wavelet-transform EXAFS (Fig. 8g). Obviously, this discovery implied that the incorporation of introduced atomic clusters influenced the electronic configuration of Fe–N sites at an atomic level. Associating chemical state measurement, Fe–N–C catalysts with attached Fe clusters, exhibiting a higher degree of Fe oxidation, elucidated enhanced efficiency in reducing the energy barrier for the ORR reaction compared to their nanoparticle counterparts (Fig. 8h), resulting in a decreased overpotential. The presence of clusters was able to induce the movement of electrons from the central metal and adjacent nitrogen atoms toward the carbon substrate. The modification gave rise to a perturbation of the electron configuration of the central metal, thereby promoting an enhanced release of OH* intermediates (Fig. 8i). The occurrence of electron redistribution around the Fe–N active site as a consequence of the interaction between nanometer-sized Fe clusters and single atoms (Fig. 8j). Concurrently, by evaluating the disparity in free energy between electronic transitions steps at 0 and 1.23 V (Fig. 8k), Fe–ACSA@NC featuring the highest Bader charge exhibited a reduced OH* binding tendency, ensuring the preservation of the active site for subsequent reactions. Furthermore, the utilization of atomic clusters to decorate M–N–C was extendable to other metals, such as Co and Ni. A comparative analysis of ORR performance indexes exemplified by Fig. 8l demonstrated substantial enhancement in catalytic performance achieved through atomic cluster decoration.

In consequence, the advances of cluster originates from its size effect, compared with nanoparticle. As active sites, cluster possesses more exposed active area. As modifier, the incorporation of clusters is beneficial to the redistribution of electronic structure, contributing to the improvement of adsorption/desorption processes for reaction intermediates and an acceleration in reaction kinetics [100, 101].

## Electrocatalytic Reactions

Till now, platinum (Pt) and other precious metal have been considered as highly efficient electrocatalyst on account of their temperate molecules binding energy. Nevertheless, the expense and limited availability of them is the biggest obstacle of large-scale applications. Therefore, the optimization of atomic-scale utilization, coordination structure and integrated construction with other effective sites take the lead in minimizing the required loading mass and maximizing their activity. A wide variety of synergetic strategies has been summarized in Sect. 3. The synergistic interaction among different components can manipulate electronic density effectively, which promotes the efficiency of interaction between reaction intermediates and the catalytically active sites in electrochemical reactions. This section will summarize the recent advancements of SACs involving various components in diverse electrochemical reactions, including HER, OER, ORR, and other reactions [35, 102–104].

### Hydrogen Evolution Reaction (HER)

**Fig. 9:**
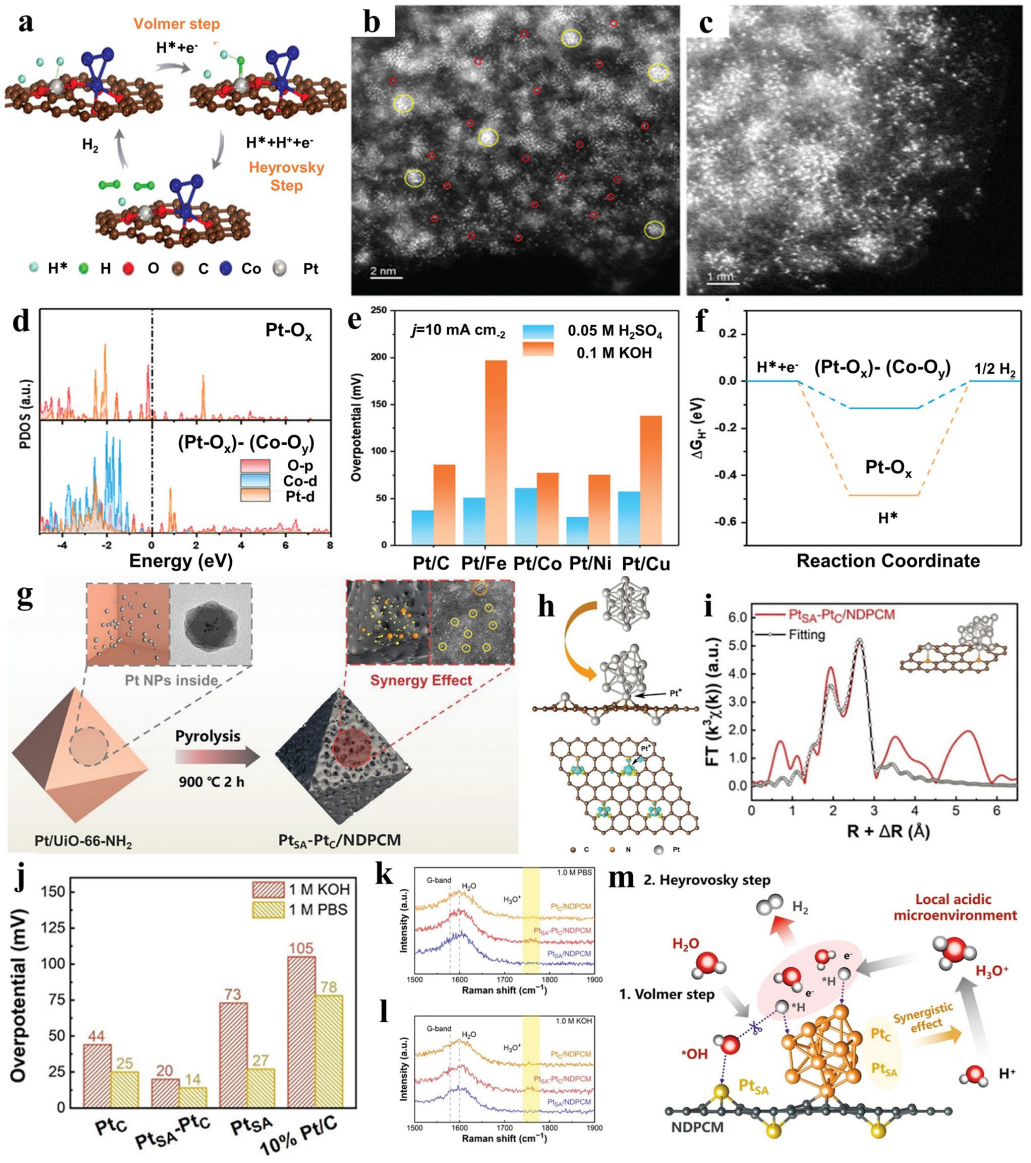
**a** Proposed HER mechanism on (Pt–O_x_)-(Co–O_y_). **b, c** HAADF-STEM images of Pt_SA_/Co_AC_-O@ACTP. **d** Diagram illustrating the difference of the PDOS of Pt, Co, and O orbitals after introducing Co cluster. **e** Overpotentials of various synergistic catalysts with different metals at 10 mA cm^−2^. **f** Calculations of Gibbs free energy for HER. Reproduced with permission [55]. Copyright 2022, Wiley–VCH GmbH. **g** Process design model for the synthesis of Pt_SA_-Pt_C_/NDPCM. **h** Atomic structures of isolated Pt_13_ clusters and the Pt_SA_-Pt_C_/NDPCM after geometrical optimization, and Charge density difference before and after Pt embedding. **i** EXAFS fitting curves of Pt_SA_-Pt_C_/NDPCM of the Pt L_3_-edge. Insert: the atomic models of the Pt_SA_-Pt_C_/NDPCM. **j** Comparison of overpotentials required to achieve − 10 mA cm^−2^ for various catalysts in 1.0 M KOH and 1.0 M PBS. Operando Raman spectra of Pt_SA_/NDPCM, Pt_SA_-Pt_C_/NDPCM and Pt_C_/NDPCM in **k** 1.0 M PBS and **l** 1.0 M KOH. **m** Schematic illustration of the mechanism in the HER processes. Reproduced with permission [112]. Copyright 2023, Wiley–VCH GmbH

As a half-reaction in water splitting, hydrogen evolution reaction plays a critical and fundamental role [31, 105–110]. Reducing overpotential, reducing the change in overpotential before and after ADT, as well as minimizing the costs associated with electrocatalysts are crucial objectives in water electrolysis. The hydrogen evolution reaction encompasses two primary steps: the adsorption of hydrogen (H) and subsequent desorption of hydrogen molecules (H_2_). The initial stage, known as the volmer reaction, entails the creation of a metal-hydrogen bond through electrochemical hydrogen adsorption. The desorption of hydrogen molecules leads to the occurrence of different mechanisms for the hydrogen evolution reaction. Figure 9a illustrates the Volmer-Heyrovsky reaction mechanism, which is a consequence of the electrochemical desorption process of H_2_. Jiao et al. [55] exploited a strategy to construct a novel nonbonding structure termed (Pt–O_x_)-(Co–O_y_), which entailed the attachment of Pt atoms and Co atomic clusters onto porous carbon derived from coal tar pitch containing high oxygen content (marked as Pt_SA_/Co_AC_–O@ACTP), to adjust the adsorption/desorption energy of H on metals for enhancing the HER performance. The aberration-corrected HAADF-STEM image (Fig. 9b, c) unveiled numerous bright spots exhibiting substantial image contrast dispersed across the ACTP support. Some of these bright spots appeared as isolated single atoms, while few atoms aggregated forming clusters in close distance. Simultaneously, Pt/M–O@ACTP containing diverse metal elements (Fe, Ni, Cu) were synthesized either and HER performance were assessed under the uniform conditions. All these catalyst variants demonstrated notably higher HER activity compared with Pt@ACTP according to Fig. 9e. These findings substantiate the pivotal role played by the Pt–O_x_ and M–O_y_ nonbonding structures in augmenting HER activity. Subsequently, relevant theoretical calculations were conducted to elucidate the electronic structure of Pt_SA_/Co_AC_–O@ACTP. The PDOS consequences revealed distinct Pt-d bands associated with (Pt–O_x_)-(Co–O_y_), as well as their positioning concerning the Fermi level (Fig. 9d). Notably, the Pt-d band of Pt-O_x_ exhibited a narrower and more distinct profile in comparison with that of (Pt–O_x_)-(Co–O_y_). Similarly, a decrease in the localization of the electronic states within the O-p band in (Pt–O_x_)-(Co–O_y_) was observed, indicating that leading nonbonding Co–O clusters into entirety regulates electronic density of Pt–O_x_. In addition, Pt-d bands validated a strong alignment with Co-d bands within (Pt–O_x_)-(Co–O_y_), facilitating a favorable electronic transfer between Pt–O_x_ and Co–O_y_. The strengthened electronic interaction led to an enhanced occupancy of *d* orbitals in close proximity to the Fermi level, potentially providing more active sites conducive to catalytic reactions. From the consequence of calculation of free energies (Fig. 9f). (Pt–O_x_)-(Co–O_y_) showed the optimal adsorption strength toward H species with ∆G_H*_ value (− 0.114 eV), which was lower than that of Pt–O_x_ (∆G_H*_ = − 0.485 eV).

In another example, Pt_SA_ and CoPt nanoparticles were encapsulated within a nitrogen-doped porous carbon framework (named as CoPt-Pt_SA_/NDPCF). In terms of the electrochemical performance, the CoPt-Pt_SA_/NDPCF electrocatalyst not only showed the only 31 mV at − 10 mA cm^−2^, but also exhibited an ultralow overpotential (199 mV) at − 300 mA cm^−2^, surpassing the commercial 10% Pt/C. Based on the aforementioned results, the catalytic performance of the CoPt alloy modulating Pt_SA_ electronic state was investigated further. The consequence obtained from Gibbs free energy analysis indicated that both Pt and Co atoms exhibited improved performance for the HER, with one or two nitrogen atoms coordination [111]. Moreover, Lian et al. [112] encapsulated single-atom platinum (Pt_SA_) and cluster platinum (Pt_C_) inside the N-doped porous carbon matrix (NDPCM) forming Pt_SA_-Pt_C_/NDPCM (Fig. 9g). According to EXAFS fitting curves connecting with more characterization data, the atomic models were constructed (Fig. 9h, i). By analyzing the variation in charge density, it was evident that there was a notable electron accumulation between Pt_SA_/Pt* atoms and substrate, signifying the existence of Pt–C bonds and robust interaction between Pt_C_ and substrate. Subsequently, Pt_SA_–Pt_C_/NDPCM exhibited remarkably low overpotentials of 14 mV in neutral conditions and 20 mV in alkaline conditions, respectively (Fig. 9j). Furthermore, the reaction mechanism of HER was investigated. From the results of Operando Raman spectra, H_3_O^+^ intermediates were detected on Pt_SA_–Pt_C_/NDPCM. However, no significant H_3_O^+^ peaks were observed for both Pt_C_/NDPCM and Pt_SA_/NDPCM (Fig. 9k, l), evidencing that the synergistic interaction between single atoms and clusters in Pt_SA_–Pt_C_/NDPCM constructed a local acidic micro-environment during the HER process. Consequently, the detailed Volmer-Heyrovsky reaction process was speculated, shedding light on the synergistic effect of Pt_SA_ and Pt_C_. The simultaneous occurrence of water absorption and desorption, facilitated by H_3_O^+^ in the acidic microenvironment (Fig. 9m), led to an augmented catalytic activity for HER under neutral and alkaline conditions.

The aforementioned examples showcase the immense potential of such catalyst in enhancing the HER performance owing to the synergistic effect among multiple active sites in it [113].

### Oxygen Evolution Reaction (OER)

**Fig. 10:**
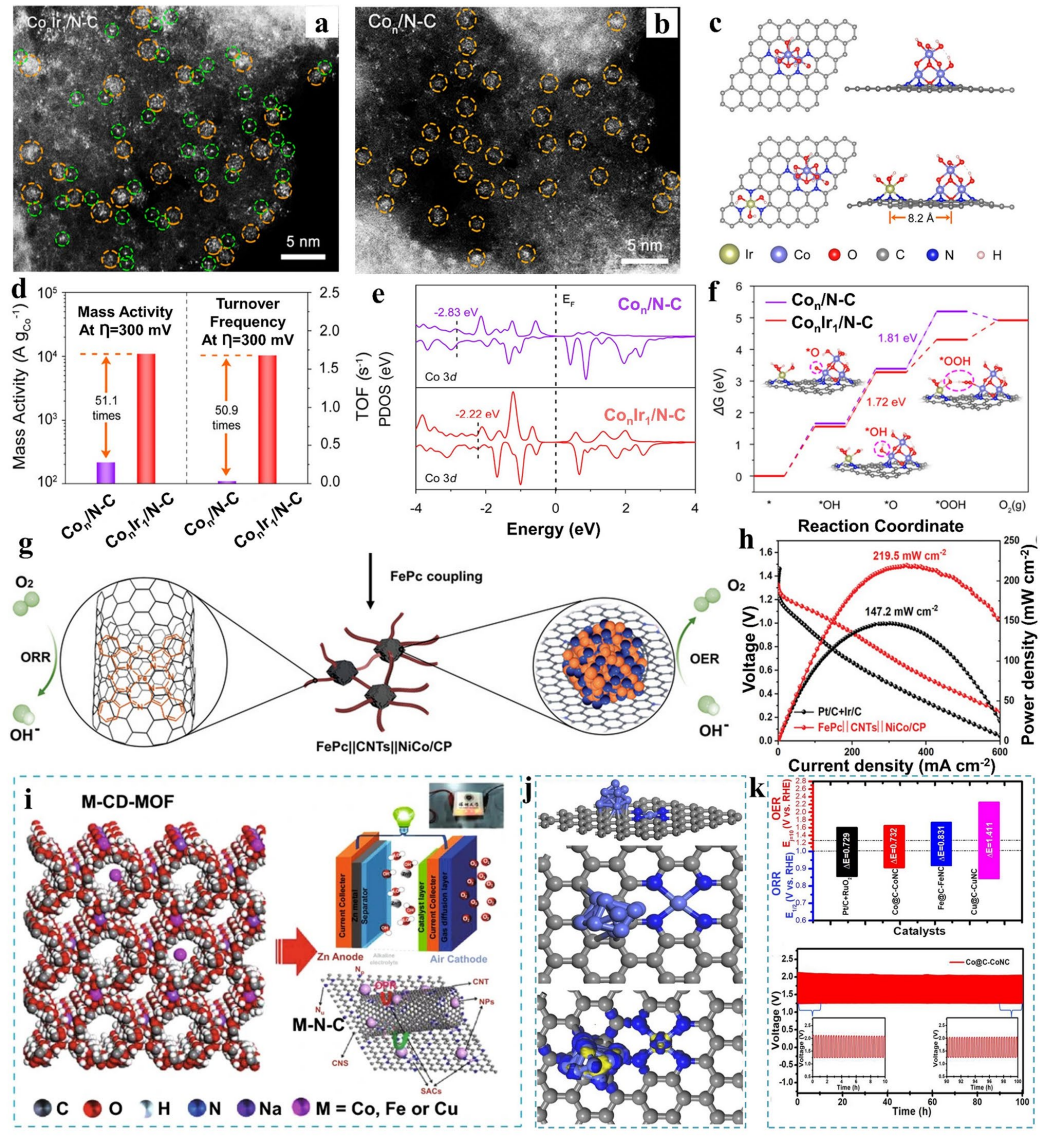
**a** Magnified HAADF-STEM images of Co_n_Ir_1_/N–C. The Co clusters and the Ir single atoms marked with different size and color circles. **b** Magnified HAADF-STEM images of Co_n_/N–C without single atomic sites. **c** Schematic structure of Co_n_/N–C, Co_n_Ir_1_/N–C top and side views. **d** Comparison of two performance indexes (MA, TOF) for Co_n_/N–C and Co_n_Ir_1_/N–C at 300 mV. **e** Projected density of states of Co 3*d* in Co_n_/N–C and Co_n_Ir_1_/N–C. **f** Gibbs free energy diagrams of Co_n_/N–C and Co_n_Ir_1_/N–C toward OER. The insets in (**f**) portray the adsorption arrangement on Co_n_/N–C and Co_n_Ir_1_/N–C at each stage. Reproduced with permission [118]. Copyright 2023, American Chemical Society. **g** Abstract diagram for components of FePc||CNTs||NiCo/CP. **h** Polarization and power density curves at 10 mA cm^−2^ of FePc||CNTs||NiCo/CP and Pt/C + Ir/C as oxygen electrodes for liquid-state ZABs. Reproduced with permission [121]. Copyright 2022, Wiley-VCHg GmbH. **i** Schematic of the M-CD-MOF, its application and reaction mechanism. **j** Atomic models with charge density difference plots for Co NPs adjacent to Co SACs in Co@C–CoNC. **k** Summarized features and realizing the best robust alkaline OER, ORR and durability performance at 2 mA cm^−2^ for ZABs using Co@C–CoNC as electrode materials. Reproduced with permission [116]. Copyright 2023, Springer

In water electrolysis devices, OER serves as the anodic reaction and proceeds via a sluggish four-electron transfer mechanism [114–117]. Enhancing the kinetics of OER is essential to reduce the overpotential associated with the overall water electro-splitting process. Conforming single atoms with clusters/nanoparticles into a unified system has been employed in OER to achieve desired catalytic performances. Bao et al. [118] came up with a heterogeneous catalyst composed of Ir single atoms and Co clusters (Co_n_Ir_1_/N–C) for efficient OER. Through the comparison of aberration corrected STEM images (Fig. 10a, b), atomic dispersion of Ir species surrounding the Co clusters was confirmed. Regarding catalysis activity, the oxygen evolution activity exhibited a remarkable enhancement on Co_n_Ir_1_/N–C with reference to single-site catalyst with only Co_n_ (Co_n_/N–C). Co_n_Ir_1_/N–C showed a mass activity (MA) of 10,989 A g_Co_^−1^ at η = 300 mV, which surpassed that of Co_n_/N–C by a factor of 51.1. Correspondingly, TOF value of Co_n_Ir_1_/N–C was determined to be 1.68 s^−1^ at the same overpotential, marking 50.9 multiples increase compared with Co_n_/N–C (Fig. 10d). Subsequently, density functional theory models of Co_n_Ir_1_/N–C and Co_n_/N–C were built to gain a comprehensive understanding of their collective influence between synergistic components on the OER (Fig. 10c). In contrast to Co_n_/N–C, the Co d-band center in Co_n_Ir_1_/N–C was shifted upwards Fermi-level with easier electron transfer (− 2.22 vs.− 2.83 eV) (Fig. 10e). Simultaneously, energy barrier for rate-determining step decreased from 1.81 to 1.72 eV as Ir species was introduced (Fig. 10f). Both the optimization of the d-band density, shifts of d-center toward Fermi-level and reduction in energy barrier elucidate synergy achieved at an appropriate distance between two synergistic components.

Another case involved the creation of a 3D hierarchical arrangement attaching Co nano-islands anchored onto Co–N–C nanosheets through an electrochemical deposition route and pyrolysis strategy [119]. When it was employed as the air–cathode, the assembled aqueous Zn-air battery exhibited a narrow deviation in voltage during charging-discharging processes (0.82 V@10 mA cm^−2^). These studies confirmed that synergistic catalyst demonstrates promotion in electrocatalysis for OER at reduced expense. Additionally, integrated synergistic catalysts containing multiple active centers emerge as bifunctional catalysts for ORR/OER. Consequently, they are widely applied as electrode materials. For instance, Huang et al. [120] synthesized ultra-stable FeCo incorporated into carbon nanotubes containing Se atoms and applied it in flexible all-solid-state Zn-air batteries as cathode and anode simultaneously. It showed an open circuit voltage of 1.405 V and a peak power density of 37.5 mW cm^−2^, significantly surpassing the performance of Pt/C + RuO_2_/C. Equally, Li and colleague [121] engineered a piece of net weaving carbon nanotubes attaching single atom Fe-active sites and NiCo alloy into one system (Fig. 10g). As it was employed in oxygen electrode, the resulting Zn-Air batteries (ZABs) performance demonstrated more substantial power density and exceptional discharge–charge durability, maintaining performance over more than 700 cycles without attenuation (Fig. 10h). This study introduced a novel approach to achieving versatile catalysts, thereby promoting the practical implementation of Zn-air batteries [122].

Moreover, Deng and co-workers [116] synthesized a variety of multifunctional M–N–C derived from M–CD–MOF incorporating single atom active sites with in-plant metal carbide (MC) nanoparticles containing various transition metal (Fig. 10i). From the models, MC nanoparticles served as electron modulators for Co–N_4_ coordination sites encompassing particles, which resulted in a noticeable alteration in their electron distribution. This modification led to an abundance of electrons, creating a highly favorable state for facilitating the oxygen reduction/evolution reaction through efficient electron release (Fig. 10j). The typical sample Co@C–CoNC exhibited exceptional performance in two half reactions, resulting in a distinguished improvement of application performance when applied in ZABs (Fig. 10k). Table 1 enumerates the synergistic catalysts utilized in Zn-air batteries. Most of them were constructed without precious metal. This renders them attractive options in further studies [123, 124].

**Table 1:** Summary of Zn-air battery performance of synergistic catalysts integrating single atom with clusters and nanoparticles in recent studies

Catalyst	Open circuit voltage [V vs. RHE]	Charge–discharge gap [V vs RHE]	Peak power density [mW cm^−2^]	Refs.
Co/Co–N–C	1.41	0.82 (@10 mA cm^−2^)	132	[119]
FePc||CNTs||NiCo/CP	1.444	0.72 (@10 mA cm^−2^)	219.5	[121]
CNT@CoSA–Co/NCP	1.45	0.51 (@5 mA cm^−2^)	172	[146]
Co–SAs/SNPs@NC	1.493	No mentioned	223.5	[147]
FeMn_ac_/Mn–N_4_C	1.46	No mentioned	207	[131]
FeCo/Se–CNT	1.543	0.878 (@50 mA cm^−2^)	173.4	[120]
CoNi–CoN_4_–HPC-900	1.50	0.82 (@10 mA cm^−2^)	116	[148]
Co–NCS-2	1.47	No mentioned	292	[149]
Fe_3_C@NCNTs	1.61	0.85 (@10 mA cm^−2^)	194	[150]
CoNP@FeNC-0.05	1.51	1.36 (@75 mA cm^−2^)	104.4	[70]
CuZn/NC	1.44	1.2 (@10 mA cm^−2^)	120.8	[151]
Fe–SAs/Fe_3_C–Fe@NC	1.42	1.18 (@10 mA cm^−2^)	158	[125]
SA Fe@ZrO_2_/NC	1.47	0.13 (@10 mA cm^−2^)	250	[152]
Co@Co_3_O_4_/FeNS-RGO	1.449	1.65 (@10 mA cm^−2^)	181	[153]
Fe_3_C–FeN/ NC	1.41	1.25 (@10 mA cm^−2^)	166	[154]
Co@N–C/PCNF	1.59	No mentioned	292	[155]
Fe_SAs+NPs_Ce_SAs+Fe-ONPs_/NC	1.55	No mentioned	240.5	[30]
FeS/FeNSC	1.43	No mentioned	256	[156]
FeSA–Fe_NC_@NSC	1.48	1.33 (@10 mA cm^−2^)	259.8	[42]

As follows, the composite catalyst compromising single atom and nanoparticles can catalyze different reactions owing to the diversity of active sites. Moreover, it will be a bifunctional catalysts choice for both cathode and anode catalyst in various vehicles simultaneously.

### Oxygen Reduction Reaction (ORR)

**Fig. 11:**
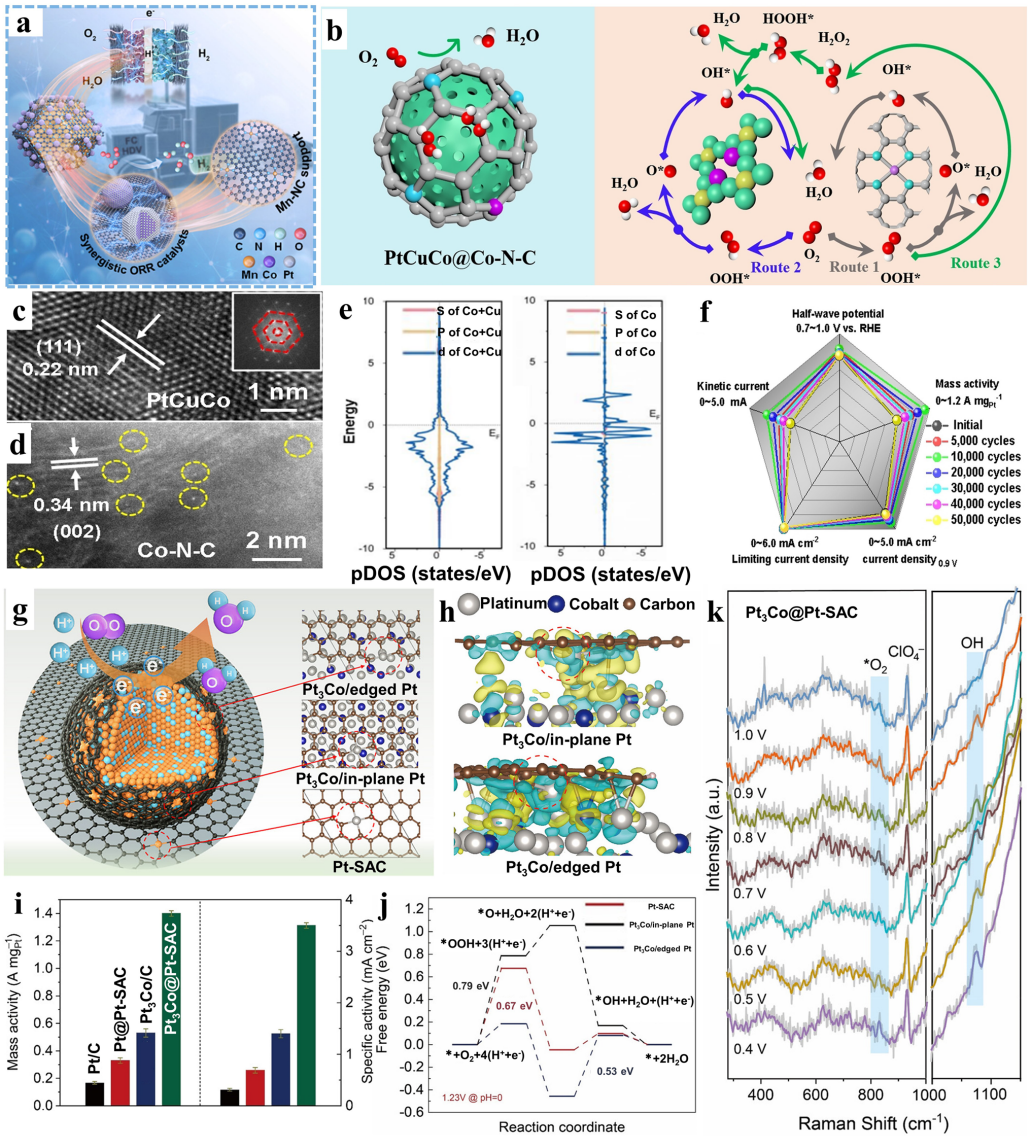
**a** Schematic diagram of Pt@Mn–N–C. Reproduced with permission [63]. Copyright 2023, American Chemical Society. **b** Analysis chart of the ORR pathways PtCuCo@Co–N–C. **c, d** HR-STEM image of alloy (insert: FFT pattern) and AC-STEM images of graphitic carbon. **e** PDOS patterns of PtCuCo alloy (left) and Co–N–C (right). **f** Activity index variation of PtCuCo@Co–N–C before and after 50,000 cycles. Reproduced with permission [130]. Copyright 2021, Wiley–VCH GmbH. **g** Diagrammatic representation for Pt_3_Co@Pt-SAC. **h** Charge density of Pt_3_Co at different orientation (Pt_3_Co/in-plane Pt and Pt_3_Co/edged Pt). **i** Catalytic performance for all samples tested at 0.9 V. **j** Free energy diagram of different catalyst models. **k** In situ Raman spectra of Pt_3_Co@Pt–SAC. Reproduced with permission [47]. Copyright 2022, American Chemical Society

In the electroreduction of O_2_, modulating the adsorption strength between catalytic centers and oxygen intermediates is the key to improve the performance. With reference to this, regulating electron density and index of d band of catalysts must be an effective way to facilitate electron transfer and procedure of bond association/dissociation (active centers to intermediates) [42, 125–129]. Typically, Wu and colleague [63] prepared Mn–N–C as carbon substrate and incorporated Pt onto it. Subsequently, the promoted synergy of composite synergistic micro-region conforming Mn single active sites with Pt in ORR was explored, enhancing intrinsic catalytic performance of precious group metal catalysts (Fig. 11a). Xia et al. [130] engineered a type of synergistic composite catalyst with the principle of multiple-scale construction brought up by themselves. They introduced graphite as substrate coating PtCuCo and single Co–N_4_ active centers, displaying enhanced efficiency in the oxygen reduction reaction. In accordance with the AC-TEM image and the corresponding fast Fourier transform pattern, a flawless crystalline structure displaying a lattice spacing of 0.22 nm was observed, revealing a strong alignment with the (111) plane of PtCuCo alloy (Fig. 11c). Additionally, graphitic carbon was demonstrated in the same way with typical lattice space index (0.34 nm), where atomically dispersed Co species were planted (Fig. 11d). Building upon the characterization outcomes, Co single-atomic site model, also including (111) plane of ternary alloy were constructed to validate the synergy in promoting ORR performance. It revealed optimized reaction pathway (Fig. 11b), while the projected Density of State (PDOS) demonstrated that synergistic composite sites possessed elevated d-band characteristics. The optimization of electron states assisted O_2_ adsorption, synchronously regulated the interaction between active site with OOH* (Fig. 11e). This synergistic composite catalyst also exhibited enhanced electrochemical performance. Some assessment criteria of ORR performance followed a consistent pattern, initially rising and then gradually declining across the entire span of 50,000 potential cycles (Fig. 11f). Besides, Liu et al. [47] immobilized platinum alloy on carbon decorated with single Pt atoms, enabling efficient and durable catalysis of the complete four-electron oxygen reduction reaction (ORR) pathway (Fig. 11g). After combination, the robust electronic interaction between synergistic components induced a change in charge density, with rich state observed between carbon support decorated with Pt sites and Pt_3_Co (Fig. 11h). The change in electronic structure contributed to the improved electrochemical performance which greatly exceeded the indexes of commercial Pt/C (Fig. 11i). Moreover, the synergistic effect in ORR was explored via DFT and In situ Raman spectroscopy (Fig. 11j, k). Specially from the in situ Raman spectroscopy, it exhibited the characteristic peak of OH*, and appeared at highest potential, suggesting the synergistic effect between Pt_3_Co and Pt-SAC.

Synergistic composite catalysts without Pt also possess immense potential in enhancing ORR performance [80]. For instance, atomically dispersed Mn–N_4_ sites were integrated with FeMn atomic clusters [131]. The resulting porous Mn atoms anchored on defect-rich nitrogen-doped carbon (Mn-DNC) derived from Mn-doped Cd-p-phenylenediamine complexes (CdMn-PPD), rich in defects, served as the host for subsequent adsorption of Fe ions. Ultimately, the hybrid catalyst was acquired through the secondary pyrolysis. Attributed to the synergy of Mn–N_4_ sites and FeMn clusters and characteristic of structure, the improved performance of the hybrid catalyst was evident, showing an enhanced ORR activity and stability. To delve deeper into the combined impact of Mn singular atoms and FeMn atomic clusters on the oxygen reduction reaction efficacy of resulting FeMn/Mn–N_4_C catalyst, DFT calculations were utilized. This resultant could be summarized that O–O bond breaking process was facilitate through the integration of single atom site and FeMn clusters, optimizing dissociation of the intermediate and encouraging four-electron oxygen reduction. Table 2 lists the ORR performances of synergistic catalysts integrating single atom with clusters and nanoparticles comparing with commercial Pt/C.

**Table 2:** Summary of electrochemical ORR performances of synergistic catalyst integrating single atom with clusters and nanoparticles in recent studies

Catalyst	E_1/2_ [V vs.RHE]	Tafel slope [mV dec^−1^]	MA[A mg_Pt_^−1^] (at 0.9 V_RHE_)	Electrolyte	Refs.
Fe_AC_@Fe_SA_–N–C	0.912	61	No mentioned	0.1 M KOH	[27]
Pt_3_Co@Pt-SAC	0.943	59	1.4	0.1 M HClO_4_	[47]
PtCo/Co–NC	0.921	74	0.7	0.1 M HClO_4_	[157]
Pt_SA_/Co_AC_–O@ACTP	0.880	87	4.2	0.1 M KOH	[55]
PCNMC-Co8Zn7	0.931	No mentioned	0.956	0.1 M HClO_4_	[73]
FeN_3_–Pd@NC NBs	0.940	51	0.919	0.1 M KOH	[69]
Cu–S_1_N_3_/Cu_x_	0.900	59	No mentioned	0.1 M KOH	[90]
PtCo@NGNS	0.950	52	1.26	0.1 M HClO_4_	[158]
Pt_1.5_Ni_1-x_/Ni–N–C	0.967	55	4.1	0.1 M HClO_4_	[78]
Pt_3_Co/Fe_4_N–C	0.95	No mentioned	1.34	0.1 M HClO_4_	[68]
Pt/(Mn–N)@C	0.928	47	0.541	0.1 M HClO_4_	[159]
10% Pt/Co–N–C	0.886	73	0.223	0.1 M HClO_4_	[133]
Pt@Ni ZIF–NC	0.902	69	1.52	0.1 M HClO_4_	[160]
Pt_3_Co/Co@Co–N–C	0.922	72.4	0.362	0.1 M HClO_4_	[77]
Pt@Co SAs–ZIF–NC	0.917	62	0.48	0.1 M HClO_4_	[161]
PtCo@CoNC/NTG	0.94	71	1.52	0.1 M HClO_4_	[48]
fct-PtCo@ Co–N–C	0.95	65	1.96	0.1 M HClO_4_	[162]
PtCo/Co–N–C	0.924	64	2.71	0.1 MHClO_4_	[163]

In summary, the integrated construction strategy is effective to obtain high-performance catalysts toward ORR. The improvement is in virtue of the optimized electronic state and synergistic effect between single atomic site and clusters/particles [132]. The exact structure of synergistic composite catalysts deepens the comprehension of reaction mechanism [133, 134].

### Other Reactions

**Fig. 12:**
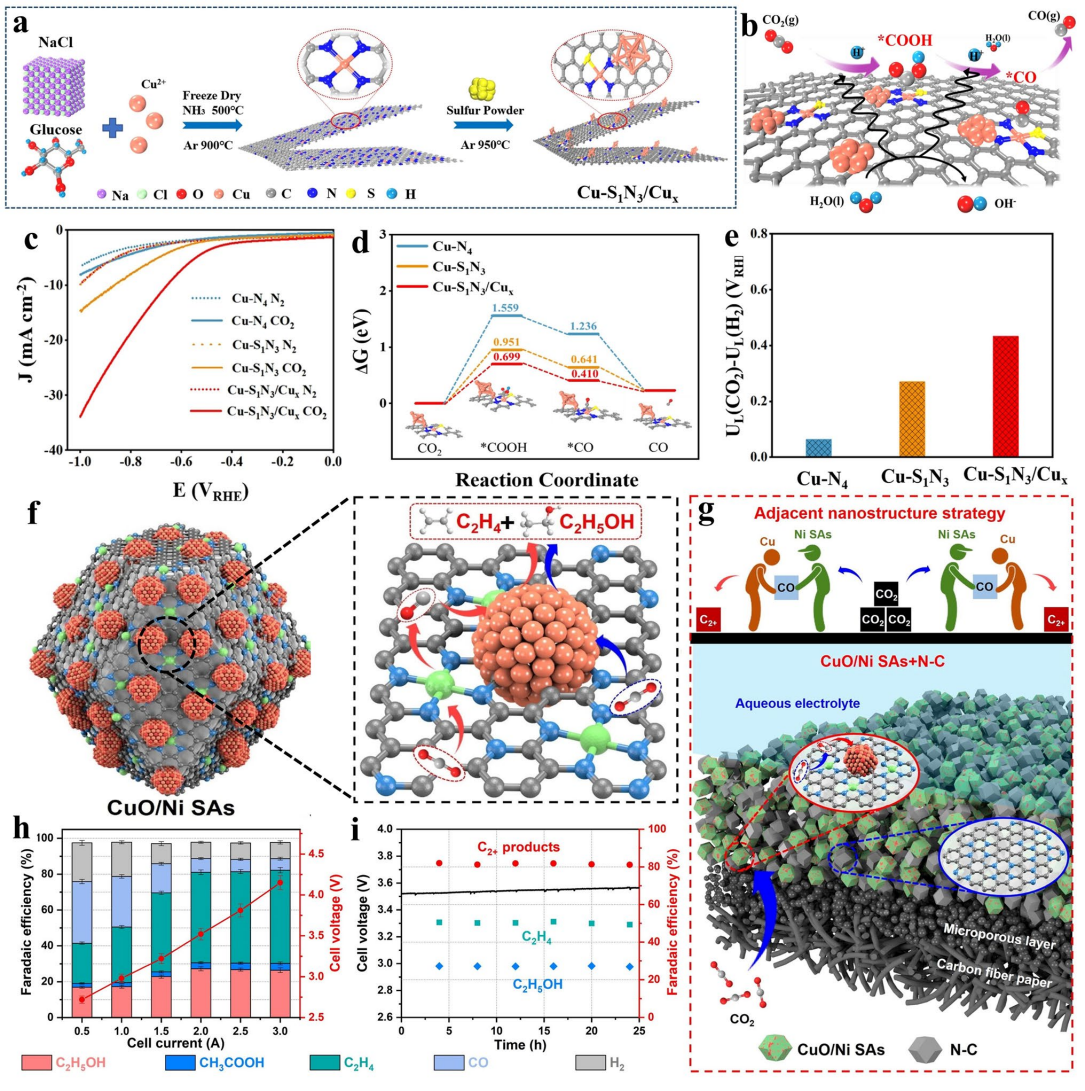
**a** Description procedure for Cu–S_1_N_3_/Cu_x_. **b** A suggested mechanism for the generation of carbon monoxide through electrochemical reduction reactions. The atom color showing in b corresponds to a. **c** LSV curves demonstrating catalytic distinction for various sites (Cu–S_1_N_3_/Cu_x_, Cu–S_1_N_3_, as well as Cu–N_4_). **d** Calculation of free-energy for the conversion of CO_2_ into CO at U = 0 V vs. RHE on three samples, respectively. **e** The limiting potential difference for CO_2_ reduction and H_2_ evolution on different catalyst models at U = 0 V vs. RHE. Reproduced with permission [138]. Copyright 2021, Wiley–VCH GmbH **f** Schematic illustrating the structure for CuO@Ni SAs. **g** Tandem catalyst electrodes fabricated with CuO/Ni SAs + N–C C_2+_ product faraday efficiency. **h** CO_2_RR performance of CuO/Ni SAs tandem catalyst in a MEA-based electrolyzer. **i** Stability of the MEA cell equipped with CuO/Ni SAs tandem catalyst. Reproduced with permission [140]. Copyright 2022, Elsevier

The reduction in carbon dioxide (CO_2_RR) has gained substantial concern over the span of years as it offers a pathway for carbon facilitating the cycling of carbon by harnessing CO_2_ [135–137]. Yu and co-workers [138] introduced an innovative tandem electrocatalyst, which applied carbon doped by sulfur and nitrogen as carrier coating Cu single atom and atomic clusters, designated as Cu–S_1_N_3_/Cu_x_ (Fig. 12a). The carbon-based catalyst showed expressively more advanced catalytic currents after introduction of Cu-based sites, especially Cu-based synergistic sites (Fig. 12c). Subsequently, DFT calculations were conducted to clarify the underlying mechanism. In Fig. 12d, it was observed that the step of transforming CO_2_ into COOH* represented the rate-determining step (RDS) without exception. The presence of the Cu cluster adjacent to CuS_1_N_3_ sites notably reduced the energy barrier to a greater extent (Fig. 12b). Besides, Cu–S_1_N_3_/Cu_x_, showed a more positive U_L_(CO_2_)–U_L_(H_2_)(V_RHE_) value (Fig. 12e). This result strongly supported the exceedingly good CO selectivity achieved by Cu–S_1_N_3_/Cu_x_. Han and colleagues engineered a novel catalyst featuring ruthenium cluster (Ru_AC_) and Ru–N_4_ (Ru_SA_) composite sites anchored onto N-doped carbon nano-box via CVD and second pyrolysis. Upon application in Li–CO_2_ cells, Ru_AC+SA_@NCB cell displays the lowest overpotentials, suggesting reduced polarization and superior electrocatalytic capability. Combining DFT with the aforementioned results, it highlighted that Ru_AC+SA_@NCB possessed higher total density, demonstrating the electronic conductivity was better [139].

Moreover, Wu et al. [140] synthesized a synergistic hybrid catalyst composed of CuO nanoparticles, as well as Ni single atoms (Fig. 12f). In particular, the neighboring nanostructure strategy tightly packed the two interactive centers (Ni, Cu), allowing independent catalysis of CO_2_–to–CO and CO–to–C_2+_, facilitating the in situ generation and rapid consumption of CO (Fig. 12g). Additionally, it was employed in a MEA-based electrolyzer. The performance index of MEA illustrated an impressive faraday efficiency of 82.7%, 52.0%, and 26.4% for the production of its highest C_2+_ products, C_2_H_4_, and C_2_H_5_OH (Fig. 12h), respectively, when operating at 3.0 A. For durability evaluation index (Fig. 12i), the cell voltage ranged from 3.521 to 3.567 V, while the faraday efficiency of C_2+_ products (82.1%), C_2_H_4_ (50.5%), and C_2_H_5_OH (27.2%) remained steady over 25 h, which presented a remarkable stability performance.

**Fig. 13:**
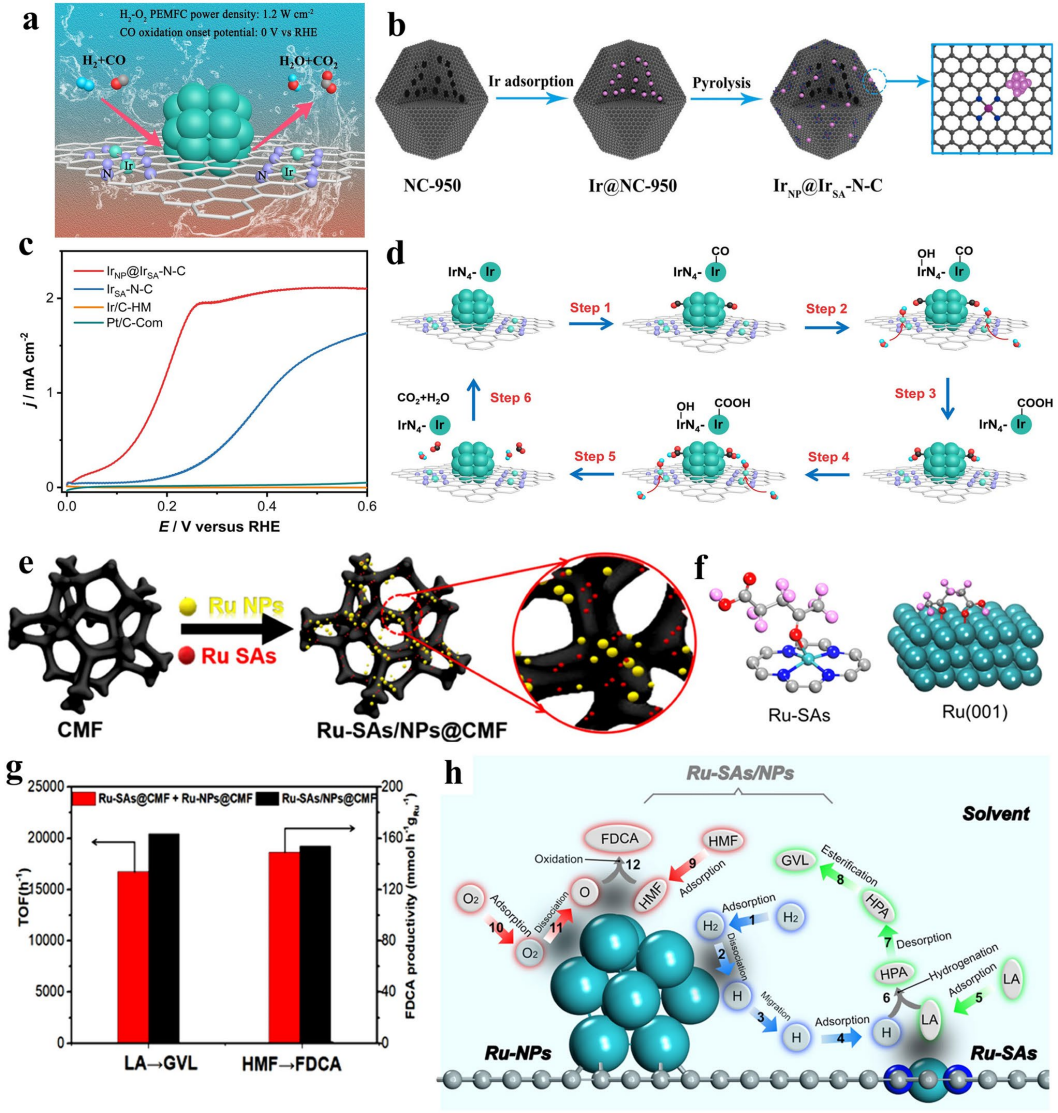
**a** Schematic for catalysis on Ir_NP_@Ir_SA_–N–C. **b** Scheme showing adsorption-carbonization procedure for the establishment of Ir_NP_@Ir_SA_-N–C. **c** Comparison of COOR performance between Ir_NP_@Ir_SA_–N–C and Pt/C catalyst in CO-saturated 0.1 M HClO_4_. **d** Schematic illustration of the optimized reaction pathway through the synergy between IrN_4_ and Ir NPs. Reproduced with permission [104]. Copyright 2021, Wiley-VCH GmbH. **e** Process flow diagram of Ru-SAs/NPs@CMF. **f** Geometric configurations of LA bonding with Ru SAs and Ru NPs. **g** Comparison of turnover frequencies for two step (LA converted to GVL and HMF oxidated to FDCA) over synergistic components and the mixture of CMF with one kind sites. **h** Diagrammatic representation of synergistic effect of two components in the Ru-SAs/NPs@CMF. Reproduced with permission [60]. Copyright 2022, American Chemical Society

Direct micro-molecule fuel cells offer enhanced energy density, while achieving complete oxidation of micro-molecule into CO_2_ and H_2_O remains highly challenging [141–144]. Xing et al. [104] fabricated Ir_NP_@Ir_SA_–N–C through a consecutive carbonization method, applied in H_2_–O_2_ PEMFC (Fig. 13a, b), subsequently utilized in CO electro-oxidation. Benefiting from IrN_4_ moiety, Ir_NP_@Ir_SA_-N–C exhibited significantly improved CO oxidation performance, demonstrating notably lower onset potential and half-wave potential verge on 0 V and 180 mV, respectively (Fig. 13c). Furthermore, the results of DFT validated that CO* adsorbed on Ir nanoparticles could be more rapidly transformed into CO_2_ on adjacent single-atom IrN_4_ only if there are reaction to generate OH*, implying that the catalyst's ability to resist poisoning was on the grounds of the synergistic effect of its components (Fig. 13d). Similarly, Qi and co-workers [103] designed a new class of synergistic catalyst integrating Pd nanoparticles and Pd single atoms toward oxidation of methanol. The synergistic effect between Pd single atoms and Pd nanoparticles induced a weak adsorption strength of poisonous intermediate species on active Pd nanoparticles to improve efficiency of methanol oxidation.

In another example, Yu et al. [145] utilized Co–N–C as support to enhance the dispersion of Pt for the aerobic oxidation of glycerol. The synergy between Co and Pt induced strong metal-support interaction and electron transfer, which was the source of enhanced oxygen activation and the overall turnover promotion. Moreover, intermetallic PtCo was integrated with Co single atom, which was confined in Pt-skin. This strategy brought this catalyst a higher d-band center and abundant electronic state, administering in activating Pt. As employed in direct ethanol fuel cell, the PtCo/Co–N–C exhibited a consistent open-circuit voltage of 0.6 V, suggesting its promising suitability for practical implementation. Meanwhile, it was noteworthy that it showcased a minor decrease in specific activity (only 6.4%) after undergoing 1,000 consecutive cycles, in contrast to the substantial 76.3% activity loss observed in commercial Pt/C under the same conditions during ethanol oxidation reactions. Therefore, decorating single-atomic site with cluster, or nanoparticles could further synergistically promote their electrocatalytic activities [141].

Additionally, this kind of synergistic composite catalyst is widely used in various reaction and application on the basis of the synergistic effect of active sites. For instance, Wang and colleague [60] engineered dual-site catalysts, Ru-SAs/NPs@CMF, which set carbonized melamine foam (CMF) derived from porous melamine foam as carbon substrate (Fig. 13e). According to the characterization, the models of Ru single sites and Ru nanocrystals were constructed. Levulinic acid (LA) was forcefully anchored through two Ru–O bonds with Ru nanocrystals, whereas in Ru–SAs, it was bonded only through one (Fig. 13f). Subsequently, comparing the TOF and 2,5 furandicarboxylic acid (FDCA) productivity of Ru–SAs/NPs@CMF to the mixture of composites that contain atoms and particles, respectively, the former exhibited superior performance, elucidating that the synergistic effects between composite sites could influence the hydrogen spillover, thereby promoting the hydrogenation of LA (Fig. 13g). Moreover, the synergistic mechanism was proposed, delineating a clear division of catalysis tasks (Fig. 13h). The construction principle of Ru–SAs/NPs@CMF has leveraged the distinctive advantages of different size sites to achieve comprehensive catalytic utilization. From these examples, not only does this composite catalysts accelerate the reaction kinetic via the synergistic effect between various active sites, but also can contrast tandem catalysis through the different characteristic of active sites.

## Summary and Outlooks

Single atoms, atomic clusters, as well as nanoparticles have been explored extensively regarding their individual activation and optimization as distinct active species. Their intrinsic catalytic activity is primarily governed by the rich partial micro-region of active centers. The surrounding and electronic state of active sites can be regulated by coupling synergistic components. The integration strategy proves to be a successful approach in enhancing the quantity of operational centers and improving the interaction between catalytic sites and support materials. Besides, the synergistic effect between these elements tunes the electron distribution, valence states, thereby optimizing the electro-catalytic performance.

**Fig. 14:**
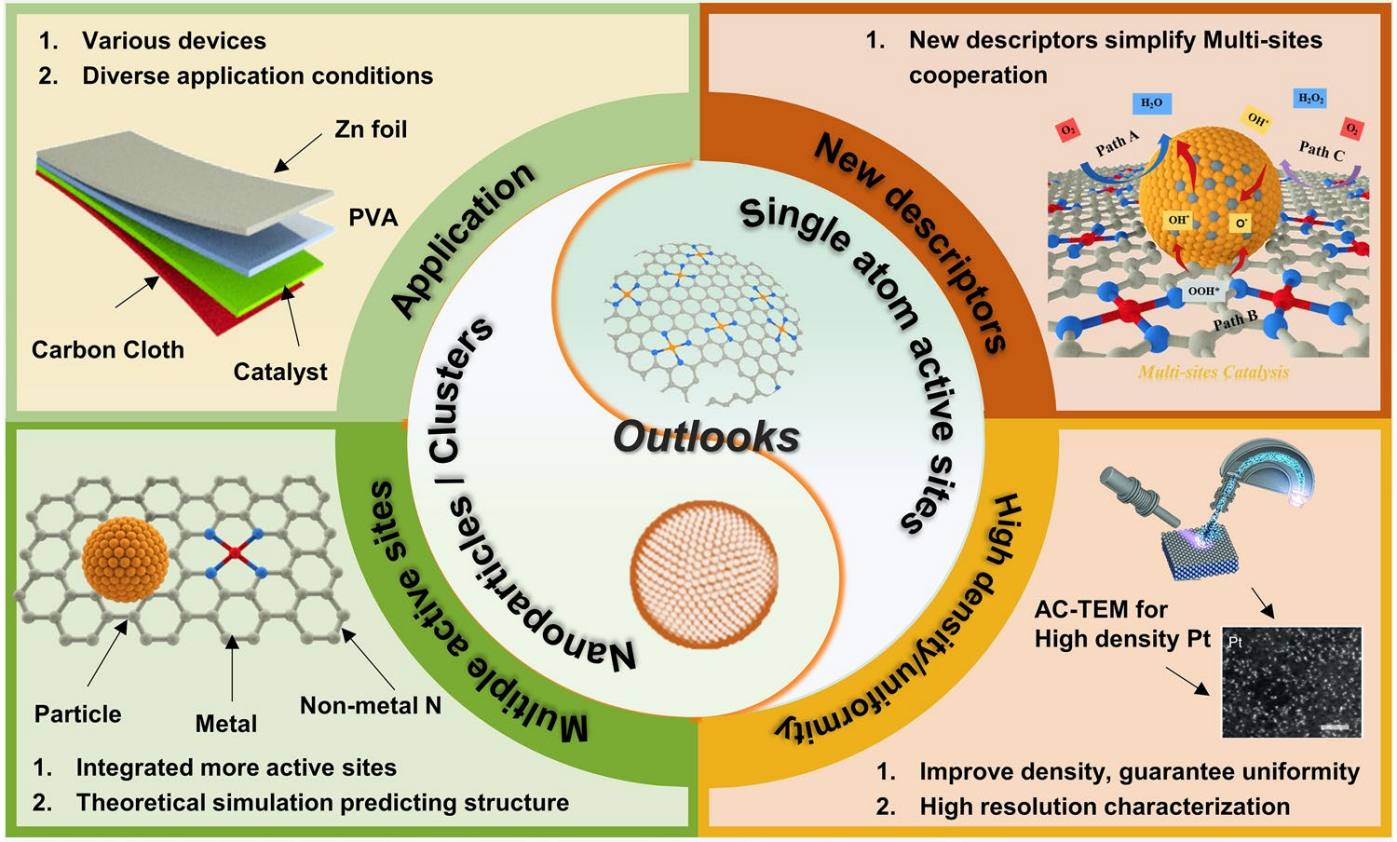
Schematic diagram of outlooks for synergistic composite catalysts

In this review, latest advances of the synergistic composite catalysts employed for electrocatalysis are overviewed comprehensively, encompassing preparation methods and characterization aimed at determining active center. Moreover, promoting dynamic nature of coupling structure is discussed, combining with their exceptional performance in energy conversion reaction, such as HER, OER, ORR, and CO_2_RR. A more comprehensive understanding is gained into the reaction mechanism via synergistic effect. Alternatively, the evolution of synergistic electrocatalysts involving various components still faces significant challenges, hindering sufficient progress. The forthcoming phase of research requires addressing numerous obstacles for advance (Fig. 14).

1. The synergistic effect of multiple active sites should be taken into consideration, especially for mutual correlation among electron configuration which will be regulated after integration. Before that, it is crucial adjective to forecast the catalytic site structure through advanced calculation, or data from machine learning. A utilization of an integration strategy enables the determination of feasible combinations of metal atoms and the identification of optimal atomic arrangements within the coupling system.
2. As we know, revealing unhindered routes for electron transfer and significantly reducing interacting distances are prerequisite for strong interaction between components. Thus, obtaining high density and uniformity can transfer from specific properties to Holistic nature. However, it is a great challenge to guarantee the uniformity while increasing the density of active sites.
3. Various active sites optimize the reaction pathway. The new descriptors could be introduced to simply the description of reaction path way. It is widely recognized that establishing a clear correlation between the chemical structure and inherent activity represents an effective approach, providing valuable insights for enlightenment of electrocatalyst design and accurate performance prediction.
4. The synergistic effect usually induces the promotion of activity, but the promotion of stability is limited. Thus, taking account into the balance of activity and stability is indispensable. Meanwhile, this composite synergistic catalyst needs to be utilized in devices in different actual application environments to study its practical application value.
